# Pollinator and floral odor specificity among four synchronopatric species of *Ceropegia* (Apocynaceae) suggests ethological isolation that prevents reproductive interference

**DOI:** 10.1038/s41598-022-18031-z

**Published:** 2022-08-13

**Authors:** Aroonrat Kidyoo, Manit Kidyoo, Doyle McKey, Magali Proffit, Gwenaëlle Deconninck, Pichaya Wattana, Nantaporn Uamjan, Paweena Ekkaphan, Rumsaïs Blatrix

**Affiliations:** 1 Plants of Thailand Research Unit, Department of Botany, Faculty of Science, Chulalongkorn University, Pathumwan, Bangkok, 10330 Thailand; 2 CEFE, University of Montpellier, CNRS, EPHE, IRD, Montpellier, France; 3 Scientific and Technological Research Equipment Centre, Chulalongkorn University, Pathumwan, Bangkok, 10330 Thailand

**Keywords:** Ecology, Evolution, Plant sciences

## Abstract

Possession of flowers that trap fly pollinators is a conservative trait within the genus *Ceropegia*, in which pollination systems can be generalized or highly specialized. However, little is known about the role of plant–pollinator interactions in the maintenance of species boundaries. This study examined the degree of plant–pollinator specialization and identified the parameters responsible for specificity among four co-occurring *Ceropegia* species with overlapping flowering times. All investigated plant species were functionally specialized on pollination by Chloropidae and/or Milichiidae flies and each *Ceropegia* species was, in turn, ecologically highly specialized on only two pollinating fly morphospecies, though one plant species appeared more generalist. Species-specific fly attraction was due to the differences between plant species in floral scents, floral morphology, colour patterns, and presence of other functional structures, e.g., vibratile trichomes, which were shown to contribute to pollinator attraction in one study species. The combination of these olfactory and visual cues differentially influenced pollinator preferences and thus hindered heterospecific visitation. Furthermore, a pollinator exchange experiment also highlighted that species integrity is maintained through efficient ethological isolation (pollinator attraction). The mechanical isolation mediated by the fit between floral morphology and size and/or shape of fly pollinators appears less pronounced here, but whether or not the morphological match between male (pollinium) and female (guide rails) reproductive organs can impede hybridization remains to be investigated.

## Introduction

Closely related plant species that co-occur in the same community and flower in synchrony, i.e. synchronopatric species, share similar habitat and may compete for both biotic and abiotic resources^1^. Synchronopatric congeners may exhibit either positive or negative interactions mediated by pollen vectors. Co-flowering species may have greater reproductive success by attracting more pollinators through larger floral displays^2,3^. Alternatively, co-flowering may lead to reproductive interference, i.e. interspecific sexual interaction that negatively affects fitness^4^ of one or more of the co-flowering species due to increased competitive interactions among plants for pollinators and the costs of interspecific pollen transfer and gametic wastage^5^. Nevertheless, these costs may be negligible when effective reproductive isolation mechanisms are in place.

Maintenance of species boundaries results from pre- and/or post-zygotic isolation mechanisms. Their relative contributions are highly variable among plant groups and depend on specific characteristics of their reproductive biology^6,7^. Asclepiadoideae (Apocynaceae) and Orchidaceae are characterized by pollen grains packaged into pollinia, often associated with a variety of accessory structures, forming a pollinarium—the functional unit (one to eight in orchids, five in asclepiads) removed by the pollinators^8^. Consequently, only one or a few visits may result in the removal of all pollen from a flower. The cost of reproductive interference through pollen loss is thus particularly high for these plants. Asclepiads and orchids are expected to be under strong selective pressure to hamper pollen loss due to pollinator sharing and interspecific pollination through pre-pollination isolation mechanisms. Indeed, these barriers have been reported in many orchids^9–11^, but have been less investigated in asclepiads^12–14^. Pollinator specificity is of primary importance in maintenance or reinforcement of reproductive barriers. Pollinator-mediated reproductive isolation may be effected by floral isolation, either mechanical (morphological fits between pollinators and flowers and/or between male and female reproductive organs) or ethological (pollinator attraction), resulting from differential visitation or constancy among pollinators driven by variable floral traits^7,12,15^. Divergent floral traits between coexisting populations of congeneric species may occur as a by-product of allopatric speciation or may result from selection for reproductive isolation per se (especially in cases of reproductive interference)^16,17^.

The genus *Ceropegia* s. str. (Apocynaceae, Asclepiadoideae) has flowers characterized by (1) gynostegium, a structure formed by fused, highly modified male (five stamens) and female (five stigmatic chambers behind the guide rails, the latter being two hardened lateral margins of anthers) reproductive organs and (2) corona, a series of appendages around the central gynostegium. As in other Asclepiadoideae, pollen is aggregated into pollinia, a pair of which is connected to a middle corpusculum by translator arms. Despite large variation in floral traits across species, the genus is functionally specialized (same functional group of pollinators exerting similar selective pressures on floral traits, often including functionally similar members of higher taxonomic groups, typically family or above)^18,19^ for pollination by small Diptera^20–22^. Most species are considered to rely on deceptive pollination, trapping flies for a fixed period of time, during which pollen removal and/or deposition occurs. The level of specialization in pollinator attraction is highly variable among species, some taxa being specialists attracting flies from a single family or genus, such as *C. sandersonii* and *C. pachystelma* that use respectively *Desmometopa* spp. (Milichiidae) and *Forcipomyia* sp. (Ceratopogonidae) as main pollinators^23,24^, and others being generalists attracting flies of various families or genera, such as *C. ampliata*, which uses flies in the families Anthomyiidae, Lauxaniidae, Muscidae, Sarcophagidae and Tachinidae^24,25^. Moreover, these associations may be conservative, i.e., constant over most or all of the plant’s native range^21^ and even when plants are transported out of their native ranges and grown in greenhouses^26^, or less conservative, wherein different pollinator assemblages are used by individual plants of the same species within different parts of their native range or outside their range^21,25,27^. To date, it is still unknown whether this variability is due to spatial variation in local pollinator communities or whether there also exist other factors or strategies influencing the degree of specificity of interactions. Likewise, the extent to which plant species affect one another’s pollination through reproductive interference is unknown. Where habitats of co-flowering *Ceropegia* species overlap, as do those of different pollinating fly species, can interspecific visitation be avoided by differential attraction to pollinators through specific floral traits? In addition to a low number of pollen transfer opportunities (only five pairs of pollinia) per flower, individual *Ceropegia* plants display very few flowers, increasing the cost of pollen loss to heterospecific flowers.

The role of volatile organic compounds (VOCs) of *Ceropegia* flowers in pollinator attraction has been extensively investigated, mostly in South African species, as olfactory signals are considered to be the most important ones for attracting flies from a distance^23,24^. Based on these olfactory cues, it has been suggested that plants exploit pollinator feeding preferences. The involvement of VOCs in prey mimicry strategies attracting kleptoparasitic flies—flies feeding on the food or prey of predatory arthropods—as pollinators has been evidenced in a small number of *Ceropegia* species^23,24^. This pollination strategy is called kleptomyiophily^28^. Chemical composition of floral scents as olfactory cues, together with floral morphology as visual cues, were found to be highly variable among species^23,24,29^. Thereby, the crucial role of these cues as ‘floral filters’ in the attraction of a limited set of pollinator species (i.e., ethological isolation)^17^ can be expected. Unlike the case for VOCs, the attractiveness of various visual cues remains undeciphered in *Ceropegia*. Among the most prominent of such visual cues are scintillant and motile structures termed vibratile trichomes (or “flickering bodies”; “Flimmerkoerper” sensu Vogel 1954, 2001)^30,31^. In addition, physical matching (i.e., mechanical isolation)^17,21,22,29^ is thought to play a certain role in maintaining species integrity. This matching involves on the one hand the fit of pollinators and flowers, which has been hypothesized to drive divergence in pollinator use, and on the other, the fit between guide rails and pollinium^32^. In *Ceropegia*, only the thin margin on the upper outer (‘outer’ relative to the anther that produces the pollinium) side of a pollinium, called the “insertion crest”, is to be inserted between the guide rails for pollination.

The region comprised by the Indian subcontinent and Southeast Asia is one of the most species-rich areas for *Ceropegia*, but information on pollination systems in this region so far remains sparse^21,33,34^ relative to other centers of diversity^21,22^. In Pha Taem National Park, located in eastern Thailand, four rare and narrowly endemic *Ceropegia* species—*C. acicularis*, *C. boonjarasii*, *C. citrina* and *C. tenuicaulis*—coexist in close sympatry. Although their rarity and their occurrence in small populations necessarily limit sample sizes, their sympatry offers a unique opportunity for studying the mechanisms of reproductive isolation among *Ceropegia* spp. The present study addresses two main questions: To what degree do the four *Ceropegia* species exhibit specialization on fly pollinators, and are pollinator assemblages species-specific? Are behavioral (floral scents) and/or morphological (floral and fly morphology) filters responsible for the observed specificity? To answer these questions, we will (1) characterize pollinator assemblages, (2) describe floral scents, (3) test for the role of visual cues (in one species that displays vibratile trichomes), (4) compare floral and fly morphometrics and (5) test for the capacity of fly species that are not natural pollinators to remove the pollinaria of a given *Ceropegia* species. As a whole, the information obtained from (2)–(5) will allow us to identify putative pre-pollination isolation mechanisms. The four species studied are endemic to the study area. We thus consider it highly unlikely that pre-pollination barriers are a mere by-product of allopatric speciation. Instead, we consider that such barriers must have been driven by selection for reproductive isolation, either ethological or mechanical.

## Results

### Pollinator assemblages are distinct among the investigated *Ceropegia* species

**Figure 1:**
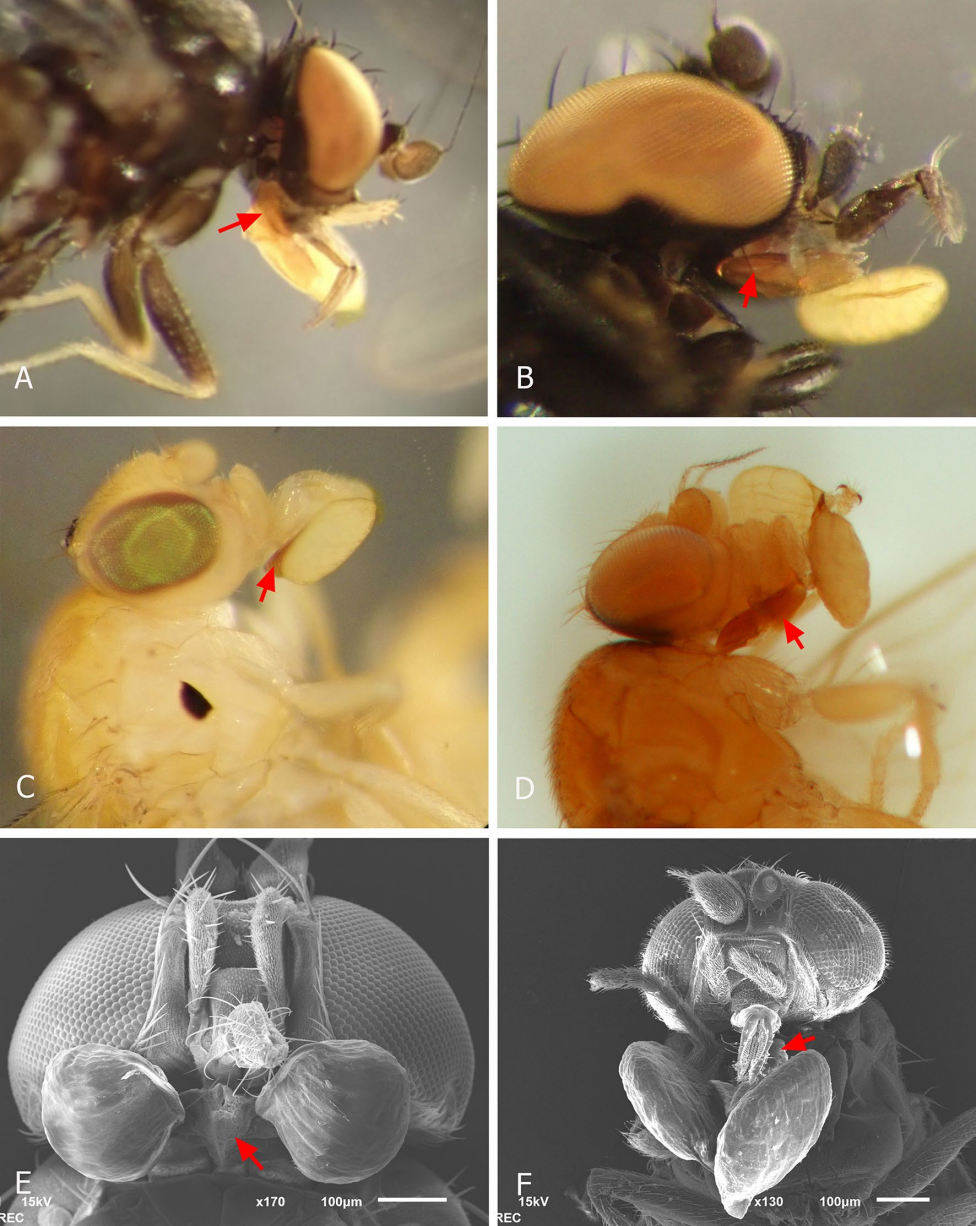
Photographs taken under a stereo microscope (**A**–**D**) and scanning electron microscopy images (**E**–**F**) of the most abundant pollinaria-carrying fly morphospecies collected from flowers of *Ceropegia* species at Pha Taem National Park, Thailand. *Neophyllomyza* sp. 1 collected from *C. citrina* flowers (**A**); *Milichiella* sp. 2 collected from *C. tenuicaulis* flowers (**B**, **E**); Chloropidae sp. 8 collected from *C. boonjarasii* flowers (**C**); Chloropidae sp. 7 collected from *C. acicularis* flowers (**D**, **F**). Red arrows indicate where on the proboscis the corpusculum of a pollinarium gets attached. Photographs by Rumsaïs Blatrix (**A**–**C**) and Manit Kidyoo (**D**–**F**).

Floral visitors found imprisoned within the tubular corollas of the four *Ceropegia* species were exclusively small Diptera. A total of 168 Diptera were collected from flowers and subdivided into 16 morphospecies using a combination of morphological and molecular analyses (Table 1, Fig. S1). All flies belonged to the families Milichiidae (42.3%) and Chloropidae (57.7%). All collected flies were female, except for two individuals of one morphospecies, i.e., Chloropidae sp. 2 (see Table 1) trapped inside flowers of *C. tenuicaulis*. Of all flies trapped within the flowers, 56 (33.3%) carried pollinaria (Table 1). All flies that were found with pollinaria attached carried them on the mouthparts, the corpuscular groove of the pollinarium being caught on the membranous posterior part of the rostrum (Fig. 1).

**Table 1:** Flies trapped in the flowers of the four *Ceropegia* species sampled in Pha Taem National Park between 2016 and 2019.

	*C. acicularis*	*C. boonjarasii*	*C. citrina*	*C. tenuicaulis*
	(n = 59)	(n = 36)	(n = 3)	(n = 20)
**Milichiidae**				
*Milichiella* sp. 1	11^(4poll)^			2
*Milichiella* sp. 2				34^(10poll)^
*Neophyllomyza* sp. 1			11^(1poll)^	
*Neophyllomyza* sp. 2			12^(2poll)^	
*Neophyllomyza* sp. 3				1
**Chloropidae**				
Chloropidae sp. 1		1		
Chloropidae sp. 2		6^(5poll)^		2 M^(1poll)^,1
Chloropidae sp. 3		2^(1poll)^		
Chloropidae sp. 4	1			
Chloropidae sp. 5		8^(1poll)^		
Chloropidae sp. 6		5^(5poll)^		
Chloropidae sp. 7	34^(12poll)^			
Chloropidae sp. 8		33^(13poll)^		1
Chloropidae sp. 9		1		
Chloropidae sp. 10		1^(1poll)^		
Chloropidae sp. 11	1			
Total flies	47	57	23	41

All flies were female, except when otherwise indicated.

*n* number of flowers sampled for each species, *M* male, ^*(#poll)*^ number of flies carrying pollinaria.

The frequencies of flies of the two families varied significantly among the four *Ceropegia* species (test of homogeneity, $\upchi$^2^ = 118.68, *df* = 3, *P* < 0.001), with *C. citrina* and *C. tenuicaulis* attracting mostly Milichiidae, and *C. acicularis* and *C. boonjarasii* mostly Chloropidae. Of the 16 delineated morphospecies, 13 were only found in flowers of a single species (Table 1); three morphospecies were present in flowers of two *Ceropegia* species, but their abundances were always much higher in one of the two species (Table 1).

Overall, there were significant differences in pollinator assemblages between the four *Ceropegia* species (PERMANOVA *F*_*3,43*_ = 14.954, *P* < 0.001). Furthermore, all the pairs of *Ceropegia* species compared differed significantly in pollinator assemblages (pairwise PERMANOVA, pairwise comparisons, *P* < 0.001 each for *C. acicularis*–*C. boonjarasii*, *C. acicularis*–*C. tenuicaulis* and *C. boonjarasii*–*C. tenuicaulis*; and *P* < 0.05 each for *C. acicularis*–*C. citrina*, *C. boonjarasii*–*C. citrina* and *C. citrina*–*C. tenuicaulis*). However, *C. tenuicaulis* was visited and/or pollinated by one morphospecies identified as a pollinator of *C. acicularis* (*Milichiella* sp.1), and by two morphospecies (Chloropidae sp. 2 and Chloropidae sp. 8) that were pollinators of *C. boonjarasii* (Table 1).

Considering the degree of specialization based on floral visitors, *Ceropegia citrina* was most specialized, i.e. on pollination by *Neophyllomyza* spp. *Ceropegia tenuicaulis* tended to be specialized on pollination by *Milichiella* sp. 2, but was also pollinated by Chloropidae sp. 2 in a much lesser proportion. Likewise, the pollination of *C. acicularis* tended to be specialized to Chloropidae sp. 7, but was also largely contributed to by *Milichiella* sp. 1. *Ceropegia boonjarasii* was most generalist, pollinated by six morphospecies of Chloropidae.

### Behavior of floral visitors

Video recordings showed flies of the families Chloropidae and Milichiidae (Fig. S2) landing on the flowers and either walking readily (for *C. acicularis*) or falling (for *C. boonjarasii*, *C. citrina* and *C. tenuicaulis*) into the corolla tube (e.g., Supplementary Information Video 1). Interestingly, most Chloropidae flies observed visiting the flowers of *C. acicularis* purposefully walked downward into the corolla tube. Moreover, some insects climbed back up and left after entering the flowers for a while. Such a circumstance, in which flies were not trapped in flowers of *Ceropegia*, has so far not been observed in other studies. Video recordings inside flowers showed flies moving downward to the bottom of the corolla where the gynostegium and corona are located (e.g., Supplementary Information Videos 2 and 3). Flies then probed the area around the gynostegium, and eventually removed or deposited pollinaria. For a full description of floral anthesis and pollinator behavior based on video recordings, see Supplementary Information S1.

The flowering of all studied *Ceropegia* species peaked in the month of July. During this period of time, the flowers were visited between 8:00 and 19:00, with different average daily peak intensity of visitation for different plant species. Two distinct patterns could be recognized. (1) Visitation (number of fly individuals per flower and hour) peaked in late afternoon when the temperature decreased to < 31 °C and the light intensity dropped to < 2000 lum ft^-2^, e.g., Chloropidae with total maximum visits to *C. acicularis* (0.9; *n* = 10) at 17:00 and to *C. boonjarasii* (2.8; *n* = 9) at 18:00, and *Neophyllomyza* with total maximum visits to *C. citrina* (3.3; *n* = 3) at 16:00. (2) Visitation peaked in the middle of the day, the period of time with the highest temperature (33–37 °C) and light intensity (5800–11,000 lum⋅ft^-2^), e.g., *Milichiella* with total maximum visits to *C. tenuicaulis* (3.3; *n* = 10) at 13:00 and 15:00, and to *C. acicularis* (0.5; *n* = 10) at 10:00. In both cases (1) and (2), the relative humidity stayed constant at around 75%.

### Pollinator exchange experiments

The flies previously collected from *C. boonjarasii* and *C. tenuicaulis* flowers, and a laboratory stain of *Drosophila melanogaster*, were introduced into *Ceropegia* flowers that they did not naturally pollinate (Table 2). As a whole, in this experiment, 28 pollinaria were removed by 41 flies that had been introduced. However, only 15 flies had pollinaria attached to the mouthparts at the end of the experiment, most of the other pollinaria being found free within the flowers (they probably fell off the flies). Nevertheless, all fly species tested, including *Drosophila melanogaster* laboratory strains, were capable of removing the pollinaria (Table 2).

**Table 2:** Scheme and results of the pollinator exchange experiment.

Recipient flower	Fly source	Fly species	No. of flies introduced	No. of flies carrying pollinaria^a^	No. of removed pollinaria which were lose inside the flower^a^
*C. acicularis* #1	Lab strain	*Drosophila melanogaster*	2	1	1
*C. acicularis* #2	Lab strain	*D. melanogaster*	4	1	3
*C. acicularis* #3	*C. tenuicaulis*	Unidentified^b^	1	0	1
	Lab strain	*D. melanogaster*	3	1	
*C. acicularis* #4	Lab strain	*D. melanogaster*	2	2	1
*C. acicularis* #5	Lab strain	*D. melanogaster*	4	0	4
*C. acicularis* #6	*C. tenuicaulis*	*Milichiella* sp. 2	1	0	0
*C. acicularis* #7	*C. tenuicaulis*	*Milichiella* sp. 2	2	2	0
*C. citrina* #1	*C. boonjarasii*	Chloropidae sp. 5	1	1	0
	*C. boonjarasii*	Chloropidae sp. 6	2	1^c^	
*C. citrina* #2	*C. boonjarasii*	Chloropidae sp. 4	1	1	1
	*C. boonjarasii*	Chloropidae sp. 6	3	2	
*C. tenuicaulis* #1	Lab strain	*D. melanogaster*	8	0	0
*C. tenuicaulis* #2	Lab strain	*D. melanogaster*	3	0	0
*C. tenuicaulis* #3	*C. boonjarasii*	Chloropidae sp. 6	4	3	1

The removed pollinaria were either still attached to a fly or loose (found free) inside the flower, unless otherwise stated by a footnote.

^a^At the end of the experiment.

^b^The fly was too damaged to be identified at the end of the experiment.

^c^The fly had two pollinaria attached to its mouthparts.

**Figure 2:**
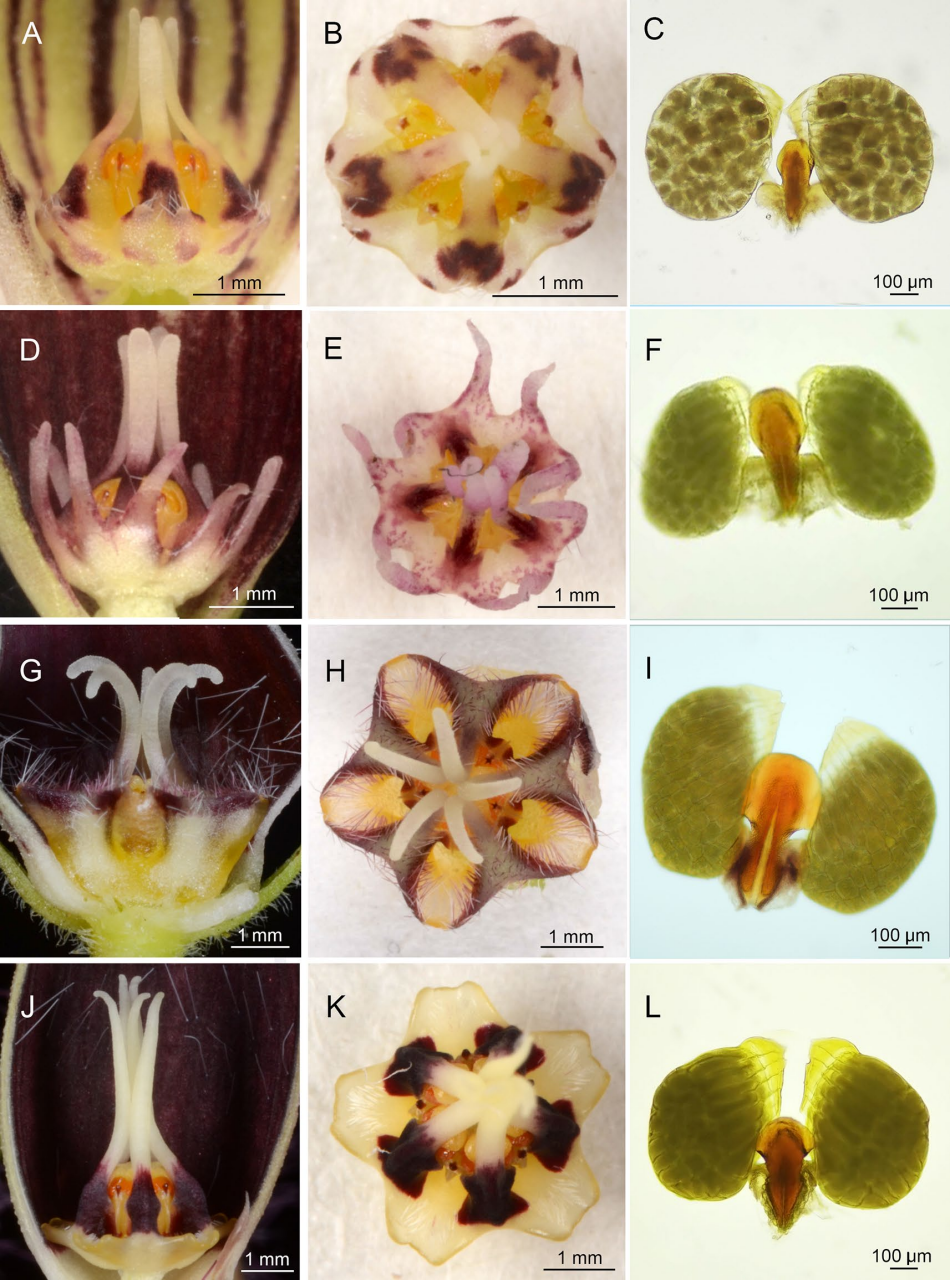
Morphology of gynostegia and coronas in side view (**A**, **D**, **G**, **J**) and in top view (**B**, **E**, **H**, **K**), and the microstructure of pollinaia (**C**, **F**, **I**, **L**) of *Ceropegia acicularis* (**A**–**C**), *C. boonjarasii* (**D**–**F**), *C. citrina* (**G**–**I**) and *C. tenuicaulis* (**J**–**L**).

The combination of shape, size and arrangement of the corona around the gynostegium was unique to each *Ceropegia* species (Fig. 2), with the result that the pollinaria and guide rails were exposed to the insect visitors in different degrees at different reciprocal positionings. Furthermore, the shape and size of pollinaria, translator arms, and especially of the insertion crests, markedly differed between *Ceropegia* species (Fig. 2).

### Morphometric analyses of flowers and flies

**Figure 3:**
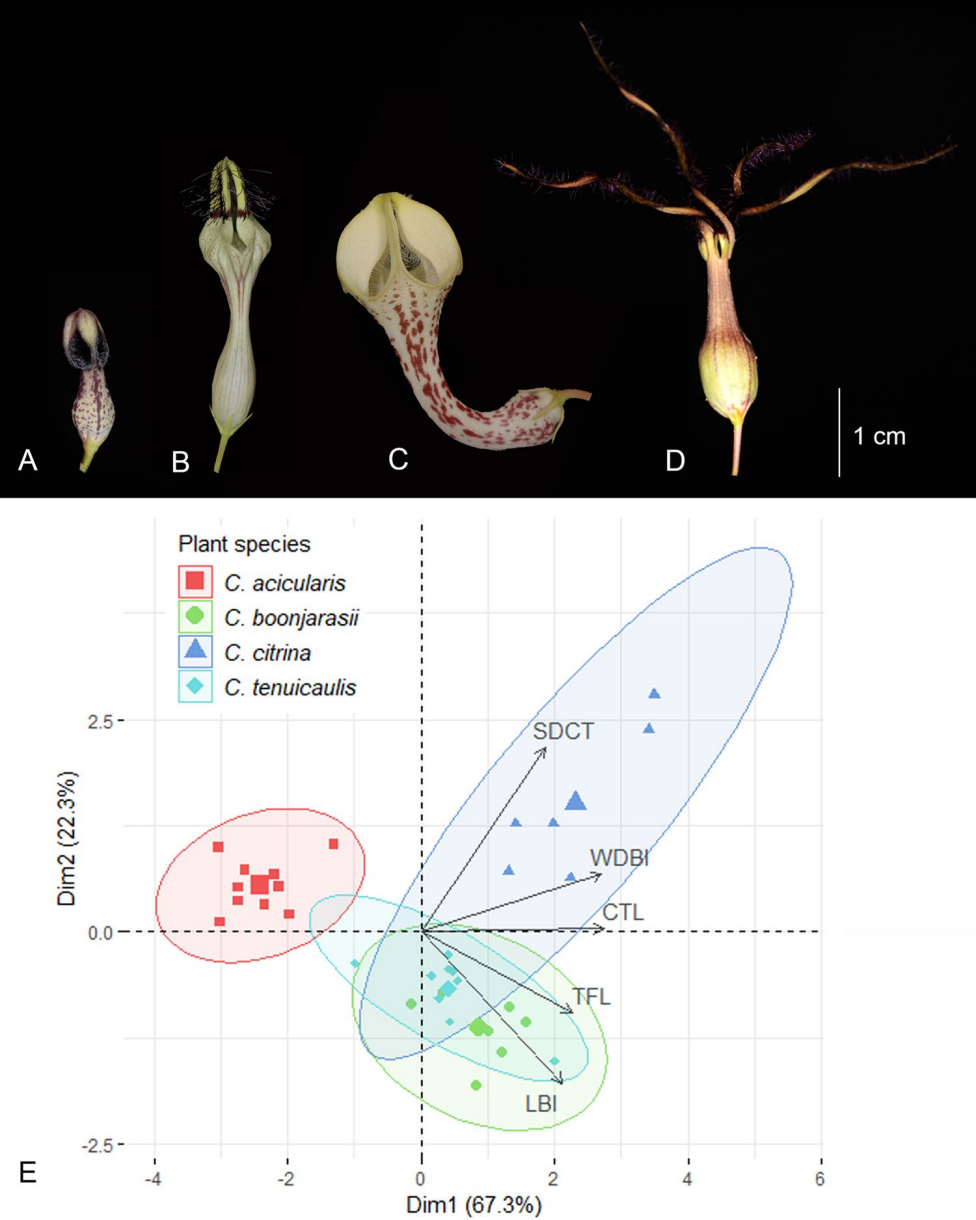
Floral morphological differentiation among the four sympatric *Ceropegia* species. (**A**) *C. acicularis*; (**B**) *C. boonjarasii*; (**C**) *C. citrina*; (**D**) *C. tenuicaulis*; (**E**) PCA biplot of individual flowers based on measurements (*TFL* total flower length, *CTL* corolla tube length, *SDCT* smallest diameter of corolla tube, *LBI* basal inflation length, *WDBI* widest diameter of basal inflation). Colored concentration ellipses are given around individuals for each species (grouping variable).

Of the four studied species, two, i.e., *C. acicularis* and *C. tenuicaulis*, had short erect stems, and flowered at 10 to 50 cm height from the ground, whereas the other two were twiners. *Ceropegia boonjarasii* climbed and flowered up to 200 cm above the ground, *C. citrina*, however, did not climb up very high and presented its flowers not higher than 50 cm above the ground. The main floral morphological differences between the four species can be clearly discerned (Fig. 3A–D). Considering the overall size of the flowers, those of *C. citrina* were largest. Also, in this species, the corolla tube was strongly curved, distinguishing it from the other species studied, all of which had a straight tube. The flowers of *C. acicularis*, with their extremely short corolla tubes, were much smaller than those of the other species. Flowers of *C. tenuicaulis* were characterized by linear lax corolla lobes with long free tail-like tips. The most distinctive feature of the flowers of *C. boonjarasii* were the clusters of long dark purple hairs that fluttered freely in the wind. Furthermore, the behavior of flowers upon withering differed from one species to another. Flowers of *C. acicularis* remained erect; those of *C. citrina* turned horizontal, so that the upper part of the curved tube was parallel to the ground; and flowers of *C. bonnjarasii* and *C. tenuicaulis* turned upside down.

Overall, each of the five floral morphological characters measured differed significantly between species (Table S1A,B, MANOVA, *F*_*3,29*_ = 50.48, *P* < 0.001). The first two axes of the PCA, explaining 89.7% of the total variance, clearly discriminated the species, except for *C. tenuicaulis* and *C. boonjarasii*, which showed substantial overlap (Fig. 3E). The floral traits that contributed most to the separation in the first and second axes were respectively the corolla tube length (26.9%) and smallest diameter of corolla tube (50.9%). *Ceropegia acicularis* and *C. citrina* were the two most contrasting in these traits. The former species had the shortest corolla tube length and the narrowest smallest diameter of the corolla tube, whereas the latter species had the longest corolla tube length and the largest smallest diameter of the corolla tube (Table S1A). The size of flies ranged from 1.4 to 2.4 mm in length and 0.5 to 1.0 mm in width (Fig. S3, Table S2A). The overall body sizes (length and width) of the different insect morphospecies differed significantly (MANOVA, *F*_*11,50*_ = 7.028, *P* < 0.001). The smallest flies measured, Chloropidae sp. 1 and sp. 3 (Table S2A), were found in the flowers of *C. boonjarasii*, which possessed the smallest tube opening (Table S1). Nevertheless, the three largest flies, Chloropidae sp. 11 and the two morphospecies of *Milichiella* (Table S2A), were found in the flowers of *C. acicularis* and *C. tenuicaulis*, which have the second and third smallest values for tube opening (Table S1). There were no significant relationships between fly size, either in length or width, and the smallest diameter of the corolla tube (Table S2B).

### Analyses of floral volatiles

A total of 37 VOCs, mostly aliphatic compounds, were detected from the four *Ceropegia* species (Table 3). Each species only had four to five main compounds, i.e., present in proportions  > 5%. These main compounds were not species-specific but their abundances varied among species. The most abundant compounds were hexyl butyrate for *C. acicularis*, isoamyl acetate for *C. citrina*, and (*E*)-2-octenyl acetate for *C. boonjarasii* and *C. tenuicaulis*.

**Table 3:** Average relative amounts of volatile organic compounds (VOCs) (mean ± SE) detected in floral scents collected from the flowers at first day of anthesis of the four *Ceropegia* species.

VOCS	RI	*C. acicularis*	*C. boonjarasii*	*C. citrinaa*	*C. tenuicaulis*	Indicator Compounds	
		(n = 7)	(n = 7)	(n = 4)	(n = 6)		
Number of compounds		13	24	24	25	IV	*P*
Percentage of specific compounds		7.7	0	12.5	16		
**Aliphatic compounds**							
Ethyl acetate	614		0.14 ± 0.17		0.19 ± 0.22		
3-Methylbutanal	654		0.32 ± 0.31	0.11 ± 0.07			
1-Butanol	676	0.16 ± 0.16			0.16 ± 0.17		
4-Methylheptane	769	0.3 ± 0.3	1.47 ± 0.98		1.06 ± 1.3		
Isobutyric acid	791				2.85 ± 3.27	0.82	0.003
Hexanal^a^	799	0.15 ± 0.07	0.37 ± 0.18		0.5 ± 0.61		
2.4-Dimethyl-1-heptene	841	1.52 ± 1.49	**18.3 ± 8.19**	0.18 ± 0.12	4.34 ± 4.66	0.87	0.002
Isovaleric acid	863				3.01 ± 3.54		
1-Hexanol	871	**9.79 ± 6.41**			2.77 ± 1.89		
Isoamyl acetate^a^	878		1.04 ± 0.53	**43.93 ± 15.5**	**10.01 ± 11.61**		
Unknown m/z: 57,43,85,71,41,99	978				0.3 ± 0.32		
Butyl butyrate^a^	996	**24.64 ± 5.37**	1.55 ± 1.84	1.39 ± 0.75	0.23 ± 0.28	0.93	<0.001
Hexyl acetate^a^	1012	1.7 ± 1.00	3.31 ± 1.98	0.27 ± 0.21	**7.24 ± 4.26**		
(*E*)-2-Hexenyl acetate	1017	0.25 ± 0.25	4.42 ± 2.27		4.37 ± 3.23		
2-Ethyl-1-hexanol	1031	0.13 ± 0.13	**8.43 ± 3.31**	1.84 ± 0.67	**8.63 ± 9.64**		
2-Nonanone^a^	1091			0.13 ± 0.05		1.00	<0.001
Nonanal^a^	1103		1.85 ± 1.29	0.91 ± 0.49	0.41 ± 0.51		
Hexyl butyrate^a^	1191	**54.04 ± 7.61**		**29.54 ± 19.17**			
(*Z*)-3-Octen-1-yl-acetate	1196	**5.39 ± 4.16**	0.13 ± 0.1		1.83 ± 0.92		
(*E*)-2-Octen-1-yl acetate^a^	1217	0.21 ± 0.16	**36.03 ± 11.51**	**5.45 ± 2.27**	**34.7 ± 13.7**		
Tridecane^a^	1300		0.12 ± 0.14	0.39 ± 0.23		0.62	0.05
Octyl butyrate	1388	1.71 ± 0.44				0.93	<0.001
(*E*)-5-Decen-1-yl acetate	1395			**11.08 ± 5.32**	0.29 ± 0.23	0.85	0.002
(*E*)-2-Decen-1-yl acetate	1407		**5.68 ± 3.62**	0.8 ± 0.38	**15.08 ± 10.3**		
Hexadecanoic acid	1969		4.63 ± 5.48	1.3 ± 0.12			
**Terpenoids**							
Monoterpenes							
6-Methyl-5-heptene-2-one	987		0.58 ± 0.26	0.53 ± 0.3			
Linalool^a^	1101		2.71 ± 3.2	0.62 ± 0.62	0.3 ± 0.34		
(*E*)-Linalool oxide (pyranoid)^a^	1177		2.43 ± 1.4	0.2 ± 0.06			
Sesquiterpenes							
(*E*)-Geranyl acetone	1453		0.27 ± 0.23	0.37 ± 0.2			
Unknown compounds							
m/z: 59, 123	1073		0.51 ± 0.37	0.18 ± 0.11	0.15 ± 0.06		
m/z: 58,73	1171		**5.06 ± 1.93**		0.39 ± 0.47	0.96	<0.001
m/z: 43,99,117,114	1471				0.46 ± 0.25	0.82	0.002
m/z: 99,155,191	1517			0.16 ± 0.08	0.23 ± 0.25		
m/z: 44, 67, 91, 134, 202, 161	1556			0.15 ± 0.11		0.71	0.017
m/z: 43, 57, 73, 91, 105, 218, 189	1771			0.17 ± 0.08		1.00	<0.001
m/z: 55, 43, 69, 83, 97, 218, 189	1882		0.4 ± 0.38	0.15 ± 0.07	0.5 ± 0.44		
m/z: 69, 43, 138, 109, 218	1899		0.23 ± 0.1	0.16 ± 0.14			

This table includes both major (relative amount > 5%) and minor compounds. Only compounds present in trace amount (< 0.1%) were not included in the analysis. The unknown VOCs are listed with important ions at various mass-to-charge ratios (m/z). *n *= number of individuals sampled; *RI *= Retention index. Indicator compounds are VOCs with a significant observed indicator value (IV). Compounds in bold typeface = major compounds with relative amount > 5%; Underlined compounds are characteristic of the respective species. ^a^Compound identity verified through authentic standard.

**Figure 4:**
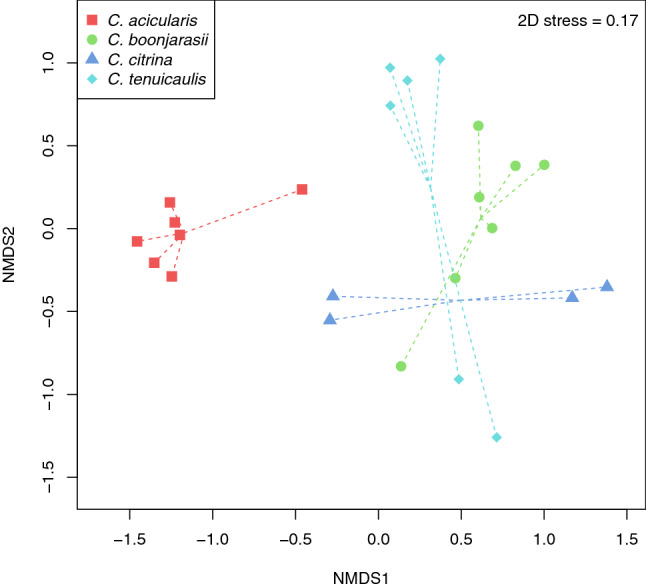
Non-metric multidimensional scaling (NMDS) based on the relative amounts of all volatile organic compounds (VOCs) detected from flowers of the four different *Ceropegia* species. The data were standardized prior to the analysis and Bray–Curtis distance was used. Only compounds in trace amount (< 0.1%) were not included in the analysis.

The VOC profiles of the four *Ceropegia* species were significantly distinct (PERMANOVA, *F*_*3,20*_ = 6.03, *P* < 0.001) as visualized in the corresponding NMDS (Fig. 4). VOCs of each of the four species were significantly different from those of the other three (PERMANOVA, pairwise comparisons, *P* < 0.05). According to the results of the indicator species analysis, some compounds could be identified as significantly representative of the odor of each species (Table 3). Among these, only three were designated as main compounds for some species: 2,4-dimethyl-1-heptene for *C. boonjarasii*, (*E*)-5-decen-1-yl acetate for *C. citrina* and butyl butyrate for *C. acicularis*.

### Attractive function of vibratile trichomes

The number of flies (mean ± SE) in unmanipulated flowers of *Ceropegia boonjarasii* was higher (5.0 ± 1.8; *n* = 7) than in flowers with trichomes removed from the corolla lobe tips (1.9 ± 0.9; *n* = 11) (GLM, *F*_*1,17*_ = 5.910, *P* = 0.04).

## Discussion

### Fly pollination in African and in Asian *Ceropegia*

The four *Ceropegia* we investigated were pollinated by Diptera of two families, Chloropidae and Milichiidae, the latter represented by two genera, *Milichiella* and *Neophyllomyza*. Pollinator assemblages were significantly different between all pairs of plant species. With regard to the pollinator specialization, there are two aspects: functional specialization, i.e. interacting with a functional group of pollinators, and ecological specialization, i.e. interacting with relatively few species of pollinators. We consider the four *Ceropegia* species in our study as equally functionally specialized since they are pollinated by Chloropidae and Milichiidae flies, both of which are long-tongued flies and often kleptoparasites. Therefore, the way these flies interact with *Ceropegia* flowers may exert similar selective pressures on floral traits. Functional specialization on pollination by Diptera of only one or two families has been generally documented in *Ceropegia*^21,22^. However, ecological specialization^18,19^ is so far infrequent in the genus. It has been shown by Heiduk et al.^24^ for other species of *Ceropegia* from East Africa and South Africa that they are not ecologically highly specialized, because the functional group of pollinators often included several fly species. A different trend is revealed here: three of the four are ecologically specialized on pollination by only two, species-specific, pollinating fly morphospecies. *Ceropegia citrina*, which only relied on two fly morphospecies of the same genus, i.e., *Neophyllomyza*, is the most specialized. In contrast, *C. boonjarasii* is most generalist, being pollinated by six Chloropidae morphospecies. Each of the other two plant species, *C. acicularis* and *C. tenuicaulis*, was pollinated by two fly morphospecies of different families. In terms of the diversity of pollinator species, these two were thus more ecologically specialized than *C. boojarasii*, but less than *C. citrina*. Both Chloropidae and Milichiidae were also reported as pollinators of *C. thaithongiae*, another Thai endemic species^33^. *Neophyllomyza* flies were found to be pollinators of *C. dolichophylla* in China^24,26^ and visitors of *C. nilotica* in southern Africa^21^, while *Milichiella* flies were visitors of *C. arabica* var. *powysii* in East Africa^21^ and *C. sandersonii* in South Africa^23^.

Large-scale studies of *Ceropegia* (including the stapeliads s. str.) covering the five major centers of diversity^21,22^, i.e., the Indian subcontinent (barring Southeast Asia), the Arabian Peninsula, East Africa, southern Africa, and West Africa, revealed flies of 16 families as pollinators. These studies also showed that the proportional use of different fly families as pollinators did not differ across all centers of diversity, thus no difference was found in this respect between *Ceropegia* from Africa and Asia. Moreover, in all regions, Chloropidae and Milichiidae were important pollinators. Note that the pollinator families used by *Ceropegia* s. str. were very different from those of the stapeliads s. str.

### Pollinator attraction and pollination strategy

A main driving force behind floral visitation by adult Diptera, which can subsequently lead to successful pollination, is their requirement for sugar as a primary energy source, which can be obtained from floral nectar, and/or nutrients necessary for reproduction, which can be obtained from pollen^35^. *Ceropegia* flowers, however, do not offer pollen that can be consumed by flies, and are generally known to be nectarless. It has been suggested that they attract fly pollinators by deception through food source or brood site mimicry^20,21,24^. Nectar has thus far only been shown to be present in an African species (*C. ampliata*^25^) and a species endemic to Thailand, *C. thaithongiae*^33^. Also, a preliminary study on *C. acicularis* and *C. tenuicaulis* revealed the presence of viscous nectar in very small volume. In both species, nectar contained about 60% of fructose and 40% of glucose (unpublished data). Despite the presence of small quantities of nectar, these species can still be regarded as deceptive since they advertise another reward via scent mimicry^21,22,24^. As *Ceropegia* flowers trap flies for several hours or days, the function of nectar has been thought to be to maintain pollinators alive until they are released^22,29,36^. In the four *Ceropegia* species studied here, oviposition has not been observed; therefore, brood-site deception is unlikely.

Recently, prey mimicry has been described in *C. sandersonii*^23^ and *C. dolichophylla*^26^. Both plant species were experimentally shown to be kleptomyiophilous, targeting their kleptoparasitic *Desmometopa* (Milichiidae) pollinators through the emission of VOCs that mimicked a particular food source for female flies. Many species of Choropidae and Milichiidae are kleptoparasites, attracted by various types of prey, frequently true bugs, killed by other arthropods. There is increasing evidence that flowers pollinated by such flies are kleptomyiophilous^22–24,28^. Typically, only females are kleptoparasitic^26,37^, because they require protein for egg maturation. The overwhelming majority (99%) of Diptera found in flowers of the four studied *Ceropegia* species were female, which is in accordance with previous findings for floral visitors of this plant group^21,22^. Forty percent of all major compounds identified in the four *Ceropegia* species are known to be released from insects, in particular by certain Hemiptera (Coreidae, Miridae, Pentatomidae). For example, (*Z*)-3-octen-1-yl acetate, (*E*)-2-octen-1-yl acetate and butyl butyrate were reported from Miridae^38,39^, and (*E*)-2-decen-1-yl acetate from Pentatomidae^40^. Hexyl butyrate, which was detected in the floral scents of *C. citrina* in a large proportion, is known to be released in prey defense secretions and responsible for the attraction of kleptoparasitic *Neophyllomyza* flies^39,41–43^ Hexyl acetate and 1-hexanol, the main compounds in the floral scents respectively emitted by *C. tenuicaulis* and *C. acicularis*, are known as components of the secretion of Coreidae bugs^44,45^. Interestingly, a species of *Milichiella*, the genus of flies that pollinated these two *Ceropegia* species*,* was reported to be attracted to various crushed Coreidae and Pentatomidae^46^. Certain VOCs have also been reported in other *Ceropegia* species, e.g., (*E*)-2-hexenyl acetate in *C. sandersonii*^23^, which is pollinated by kleptoparasitic *Desmometopa* flies and also visited by *Milichiella* flies. *Aristolochia rotunda* (Aristolochiaceae)^28^, a species with trap flowers similar to those of *Ceropegia*, emits volatiles similar to those of freshly killed Miridae bugs that were shown to attract its pollinating kleptoparasitic Chloropidae flies. These components included, inter alia, hexyl butyrate and octyl butyrate, which were present in *C. acicularis*, whose main pollinators we have also shown to be Chloropidae flies. Pollination by kleptoparasitic flies and emission of compounds also released in insect secretions support our assumption that, like other *Ceropegia* spp., *C. acicularis*, *C. boonjarasii*, *C. citrina* and *C. tenuicaulis* are kleptomyiophilous, and it is likely that VOCs play a major role in attracting their dipteran pollinators from a distance. In our study, a clear distinction in both the chemical profiles of VOC emissions of the four *Ceropegia* species and the identities of pollinating flies attracted to them suggests that each of them, if truly kleptomyiophilous, mimicks a different model. Further investigation should determine which compounds actually attract the particular pollinating flies, and which insects (models) are potentially mimicked by each species.

In addition to VOCs, which appear to play a key functional role, morphological attributes, such as shape and size of floral parts, colors and patterns or other functional traits of flowers, which were very distinctive between the investigated *Ceropegia* species, may also affect the attractiveness of flowers. The experiments performed here on *C. boonjarasii* support the hypothesis that the vibratile trichomes on the corolla lobe tips play a role in attracting the fly pollinators of this species. In their natural habitats, *Ceropegia* generally grow close to the ground, hidden among the tall grasses or in small bushes^47^. It has therefore been suggested that olfactory cues are more important than visual cues in pollinator attraction^20^. However, in the case of the twiner *C. boonjarasii*, which climbs higher up in the vegetation and can present its flowers at a level up to 200 cm above the ground, the visually attractive vibratile trichomes of its flowers appear well adapted to its growth habit and the place where it grows, playing a key role in short-distance attraction, as proposed by Vogel^20^. Similar motile floral appendages^48^ are known in other Asclepiadoideae, including *Stapelia* spp. (Ceropegieae)^49^, and in plants of unrelated angiosperm families, e.g., *Trichosalpinx* spp. (Orchidaceae, Pleurothallidinae)^50^, ﻿pollinated by Diptera^28,31,50^.

Our video recordings of flies trapped inside the flowers of *C. tenuicaulis* (Video 2) and *C. acicularis* (Video 3) are the first such recordings for *Ceropegia* trap flowers and showed that flies actively moved downwards to the base of the corolla inflation. This has been described by Vogel^20^ as positive phototaxis due to lighter-colored epidermal cells (“light windows”) around the gynostegium that direct the flies to move into contact with the reproductive organs, where they then remove pollinaria (the corpusculum being attached to the insect’s proboscis) or deposit pollinia (the guide rails take up the insertion crest of a pollinium). This uniform placement of pollinaria on different regions of insect mouthparts (e.g. rostrum, labellum or trichomes on lip pads) has been shown to be a phylogenetically conserved trait in the entire tribe Ceropegieae, which use flies, wasps, or beetles as pollinators^20–22,29,49^. In this regard, nectar (known to be present in certain species, see above) or probably minimal amounts of sugar-containing secretion contained in the cups formed by the corona appendages around the central gynostegium, may play a role in positioning the pollinators correctly.

Entrapment of flies by the tubular corolla and guidance of trapped flies to the floral reproductive organs by the light windows are the important mechanisms ensuring successful pollination in *Ceropegia*. The circumstance observed on *C. acicularis*—most of whose Chloropidae pollinators moved purposefully downward into the corolla tube, but often left the flower after a short while—implies that the flower of this species is less efficient in trapping flies. Interestingly, the main pollinators of this species preferentially visited its flowers at the end of the day, when the light intensity had decreased drastically. Therefore, once flies entered the flower, whose opening at the top of the corolla tube (i.e., the fly’s exit) was already obscured by the darkness outside, their directional movement in response to light was impeded, forcing flies to stay overnight inside the flower. Whether these circumstances reflect coincidence or alternatively specialization for pollination by flies that seek overnight shelter, they seem to mitigate the effect of inefficient entrapment of flies by flowers of *C. acicularis*.

### Reproductive isolation

The four species of *Ceropegia* under consideration were found in the same habitat with individuals of different species occurring close to each other, and had largely overlapping flowering phenology. All species seem to share the same pollination strategy and attracted flies of only one or two families (Chloropidae and Milichiidae) with similar ecological traits. Their flowers were receptive all day long, and visits of fly pollinators, although varying according to the *Ceropegia* species they visited, largely overlapped. Overall, the combination of these factors might lead to pollinator sharing and, in turn, to interspecific pollinia transfer. Hereof, Meve and Liede-Schumann^51^ suggested that hybridization mediated by small flies was most likely responsible for morphological transitions between the taxa of *Ceropegia* s. str. and those of *Brachystelma* s. str. (recently merged into the former). Moreover, one of the authors (M. Kidyoo, unpublished data) found putative hybrids in various *Ceropegia* populations across Thailand. Thus, natural hybridization in the genus is known from co-occurring species. Nevertheless, in continued observations from 2014 to 2019, no putative hybrid plant individuals (i.e., with vegetative and floral morphology intermediate between the four studied species) have ever been found in Pha Taem National Park, indicating that species integrity is well maintained through efficient reproductive isolation among the four species. Whether or not post-zygotic reproductive isolation occurs, we expect pre-zygotic isolation mechanisms to have evolved because of the relatively high value of each pollinarium (only five per flower and few flowers per plant).

The four species have diverged over time in particular floral traits. There are sharp floral morphological differences in shape, color patterns and other functional traits such as vibratile trichomes, which were shown to contribute to pollinator attraction in *C. boonjarasii*. Some similarities between the VOCs emitted by flowers of the four species suggest that the identities and proportions of compounds have changed over time due to selection exerted by different flies at this locality, which are all likely to be kleptoparasites but with different preferences for certain volatiles. Overall, specific pollinator attraction mediated by olfactory and visual floral cues—‘ethological isolation’—is a crucial pre-zygotic isolation mechanism to avoid interspecific visitation and pollinator limitation due to sharing. A contrasting case has been found in two other *Ceropegia* species, each native to different geographical regions, *C. sandersonii* from South Africa and *C. dolichophylla* from China. In that case, there is thus no selection pressure for divergence. In their natural habitats, each species uses different floral scent profiles to attract fly pollinators of the same genus, i.e., the kleptoparasitic *Desmometopa* spp. It has thus been proposed that these two *Ceropegia* exploit different olfactory preferences of flies of the same genus^23,24,26^.

Pre-zygotic isolation can also be achieved through ‘mechanical isolation’^17^, requiring morphological fit either between flower and pollinator and/or between male (pollinium, in particular insertion crest) and female (guide rails) reproductive organs, known as a lock-and-key mechanism^52^, which can be achieved through various morphological adaptations. The role of floral proportions in regulating the fly sizes that can function as pollinators has been suggested in *Ceropegia* taxa^21^. It has also been highlighted in a stem-succulent, open-flowered stapeliad, *Orbea lutea* subsp. *lutea*, the head width of whose effective pollinating *Atherigona* (Muscidae) flies is small enough to fit and probe the cavity formed by the inner corona lobes beneath the guide rails for nectar^53^. In the present study, the size of the floral entrance, i.e. the smallest diameter of the corolla tube, was thought to be the first filter screening out visitors of the four *Ceropegia* flowers. However, no effect of corolla tube diameter on the fly sizes was detected. Each fly morphospecies that pollinates one of these four *Ceropegia* species was small enough to enter the tubular corolla of the other co-occurring species. Moreover, the pollinator exchange experiment performed in this study demonstrated that, in spite of distinctive corona structure (shape, size and spatial arrangement of corona lobes) between the investigated *Ceropegia* species, which concealed to varying extents the central floral reproductive organs, all fly morphospecies tested, including flies that were not natural pollinators (even a laboratory strain of *D. melanogaster*, a widespread Diptera of a family completely different from the identified pollinators), were able to remove the pollinaria, even those of *C. citrina* that appeared to be well hidden (Fig. 2G–I). Therefore, corona structure seems not to restrict visitors from removing the pollinaria. The fact that some removed pollinaria fell off and were found free inside the flowers shows that the pollinaria were not firmly attached to the experimental non-natural visitors. It is thus likely that their micromorphology did not fit properly that of the plant’s reproductive organs. Therefore, non-specific flies can lose pollinaria in flowers and are unlikely to be able to actually insert pollinia. Whatever the fate of the removed pollinia, they are lost from the flower if they do not reach another flower of the same species. The fact that pollinaria can be removed by flies that are not naturally attracted to and visiting flowers suggests that there is selective pressure for efficient ethological isolation. The distinct floral scents among *Ceropegia* species evidenced in this study might be the result of such selective pressure.

Another aspect of mechanical isolation is the lock-and-key relation between the guide rails (lock) and pollinium (key) that has been reported from several Asclepiadoideae, e.g., *Cynanchum* s. l. (former *Sarcostemma*)^54^ and the stapeliads^32^. Other studies, however, confirmed the lack of such a precise mechanism, e.g., in *Asclepias*^55^ and *Vincetoxicum* s. l. (former *Tylophora*)^52^. Here, the preliminary study of morphology of pollinaria showed strong differences between the studied *Ceropegia* species in the shape and size of corpuscula, translator arms and pollinia, especially the insertion crest, which is the portion that is inserted as a “key” between the guide rails (the “lock”). This result is suggestive of a possible lock-and-key mechanism.

Although this first study has not yet reached a full conclusion about the mechanisms enforcing the reproductive boundaries between the four *Ceropegia* species, it advances our understanding of how these investigated species maintain their integrity, and highlights the crucial role of ethological isolation. Other core elements of work need to be done to exactly examine the risk of interspecific pollinia transfers and hybridization. On the one hand, the morphological fit between the guide rails and pollinia has to be inspected by a detailed micromorphological study. On the other, further field experiments must be conducted to test if a pollinator of a given plant species, e.g., *C. acicularis*, can insert pollinia of this plant into the guide rails of any of the other three ‘wrong’ *Ceropegia* species (and all possible combinations thereof). A simpler experiment would test if those fly species that are attracted to more than one species (e.g., *Milichiella* sp. 1) are actually capable of exporting pollen to flowers of the wrong species.

## Conclusions

We revealed that, conforming to what is known from the other centers of diversity of the genus, the four co-occurring species of *Ceropegia* endemic to Pha Taem National Park are likely kleptomyiophilous and functionally specialized to kleptoparasitic flies of two families, i.e., Chloropidae and Milichiidae. However, three of the four studied *Ceropegia* species are further ecologically specialized for pollination by only two fly morphospecies. Also, pollinator assemblages were species-specific, which could be explained by specific pollinator attraction (i.e., ethological isolation) mediated by VOCs and by floral morphology that hinders pollinaria loss due to interspecific fly visitation. In addition, these findings validate our initial hypothesis that limited opportunities of pollen transfer have driven the evolution of pre-zygotic isolation. Selective pressure drove divergent floral traits, resulting in fine-tuned attraction of specific pollinators. Floral traits resulted from local adaptation of the plant community to the fly community. Il was experimentally demonstrated here that the mechanical isolation related to the fit between flower and fly pollinator appears less effective than differential attraction by VOCs in enforcing reproductive isolation, but whether or not the close match between male (pollinium) and female (guide rails) reproductive organs can hamper interspecific hybridization remains to be investigated. Overall, the additional information obtained here for the *Ceropegia* species endemic to Southeast Asia reinforces the overall concept of pollination systems of taxa of this genus from other geographical regions, and supplies several missing pieces in the understanding of floral evolution driven by Diptera, evolutionary radiation, and reproductive isolation mechanisms responsible for the maintenance of species integrity in an assemblage of congeneric plants.

## Methods

### Study site and species

Research was conducted in Pha Taem National Park, Ubon Rachathani Province, eastern Thailand (15°23′56ʺ N, 105°30′27ʺ E, 220 m a.s.l.) (Fig. S4A,B), where four *Ceropegia* species co-exist, i.e., *C. acicularis*, *C. boonjarasii*, *C. citrina* and *C. tenuicaulis* (Fig. S5A–D). All species are endemic to the park, growing in sandy soil in the open areas of dry deciduous dipterocarp forest (see Figs. S4, S5 for more details on study site and species)^47,56,57^. The main study area was located near the national park’s headquarters. This area hosts respectively about 50, 45, 30 and 10 individuals of *C. acicularis*, *C. tenuicaulis*, *C. boonjarasii* and *C. citrina*, which occur in small patches overlapping each other (Fig. S4B). For *C. acicularis*, additional data on flower visitors were also collected at two other areas, i.e. Soi Sawan and Saeng Chan waterfalls, 10–15 km away, each with about 20 individuals. The monitoring of flowering times during 2016–2019 showed that the four species flowered synchronously from June to August. All species had peak bloom time in July. In certain years, sporadic flowers were still found until October. The details on when exactly the data were collected are provided in the relevant study methods. When the plants were in bloom, *C. citrina* and *C. tenuicaulis* only produced a single flower at a time, while the other two species regularly had several flowers open simultaneously with a maximum of six in *C. acicularis* and three in *C. boonjarasii*.

Necessary collecting permits were obtained from the Department of National Park, Wildlife and Plant Conservation (TS0907.4/12320). All the experiments conducted were in compliance with relevant institutional, national, and international guidelines and legislation. For each studied *Ceropegia* species, voucher specimens were deposited at BCU (see Supplementary Information S3).

### Flower visitors

A total of 118 samples of *Ceropegia* flowers (59, 36, 3 and 20 for *C. acicularis*, *C. boonjarasii*, *C. citrina* and *C. tenuicaulis*, respectively) were collected in 70% ethanol in the day during 2016‒2019. Although most sampling was done in July, sampling time varied year by year and covered the full flowering period. All Diptera found inside the flowers were identified to family or genus level and grouped to morphospecies using taxonomic keys^58,59^. Molecular analyses (COI sequencing) were used to further refine the delineated morphospecies.

For molecular analyses, COI sequences of 1–12 individuals (41 in total) per defined morphospecies were sequenced (24% of all collected Diptera specimens). Four morphospecies (see Fig. S1) were not sequenced due to amplification failure. DNA was extracted and COI sequences were amplified using REDExtract-N-Amp PCR Kit (Sigma-Aldrich, St. Louis, MOCR, Kit. Ref. No.: XNAT-1000RXN) and the primer pairs LCO-1490 (5′-GGTCAACAAATCATAAAGATAT TGG-3′) and HCO-2198 (5′-TAAACTTCAGGGTGACCAAAAAATCA-3′)^60^; PCR conditions were adjusted to optimize the yield. The obtained DNA samples were sequenced (both directions) at Eurofins Genomics (Germany). All specimens were kept intact and stored in the collections of A. Kidyoo (Chulalomgkorn University, Bangkok, Thailand) and R. Blatrix (CEFE, Montpellier, France) for further reference. PhyML 3.0 online (http://www.atgc-montpellier.fr/phyml/)^61^ was used to construct a Maximum Likelihood phylogeny based on which distinctive morphological traits were described for the resulting clades. A concise identification key for the defined morphospecies was provided in Supplementary Information S2. All DNA sequences obtained during this study were deposited on NCBI GenBank under the accession number OK138486–OK138526.

Each fly was checked for attached pollinaria under a stereo microscope. Afterward, a part of the fly samples was processed for observing the attachment of pollinaria on their mouthparts under a scanning electron microscope (SEM). Insect samples were dehydrated in a graded ethanol series, then subjected to critical-point drying using liquid CO_2_, sputter-coated with gold and examined using a JEOL JSM-6610LV scanning electron microscope (JEOL Ltd., Tokyo, Japan), at an accelerating voltage of 15 kV.

Body length and width of the major pollinators were measured. To determine whether frequency counts of Diptera belonging to different families were distributed identically across the four *Ceropegia* species, a chi-square test of homogeneity was performed. Differences in Diptera assemblages between the studied plant species were tested using permutational multivariate analysis of variance (PERMANOVA; 10,000 permutations) based on standardized numbers of flies. Of the total flower samples collected, only those with trapped flies inside were included in this analysis, i.e. 44 samples (12, 15, 2 and 15 for *C. acicularis*, *C. boonjarasii*, *C. citrina* and *C. tenuicaulis*, respectively), and each flower was from a different plant individual. Pairwise PERMANOVAs were run and p-values were adjusted for multiple comparisons using the false discovery rate (FDR) method^62^.

### Behavior of floral visitors

Temporal patterns of flower visitation and behavior of pollinators were recorded during 29–31 July 2017 and 14–19 July 2019 with NV-GS75 digital cameras (Panasonic, Matsushita Electric Industrial Co., Ltd., Japan) on the flowers on the first day of anthesis of *C. acicularis* (n = 10), *C. boonjarasii* (n = 9) *C. citrina* (n = 3) and *C. tenuicaulis* (n = 10) from 06:00 to 18:00 continuously. Flowers of *C. boonjarasii* were still visited by Diptera after sunset; for this species video recording time was extended for an additional 12 h (18:00 to 6:00). Visitation rates were counted from video recordings whereby only flies that entered the flowers were recorded. Identity of Diptera was recorded at family, or whenever possible, genus level. Video recording time was accompanied by recording of relative humidity, light intensity and temperature using data loggers (iButton DS1923, Maxim Integrated, San Jose, California, USA; HOBO Pendant Temp/Relative Light Two Channel and HOBO U23-001A Temperature/Relative Humidity Data Logger, Onset Computer Corporation, Bourne, Massachusetts, USA).

In addition, behavior of the insects confined inside the basal inflated portion of the flowers of *C. acicularis* (n = 3, with 3 to 5 h of observation per flower) and *C. tenuicaulis* (n = 6, with 1 to 2 h of observation per flower) was recorded by inserting the tip of a commercial mini camera into the corolla tubes cut transversely just above the basal inflation.

### Pollinator exchange experiments

In order to investigate morphology-based pollinator specificity (mechanical fit between pollinator and floral morphology), Diptera collected from flowers of one *Ceropegia* species were manually introduced into flowers of another species (Table 2). Effectiveness of an insect in pollinating flowers depends on its capacity to remove the pollinaria and to execute insertion of pollinia. Due to limited numbers of the flowers at the proper stage and of Diptera collected beforehand, the experiment was focused on the first step of pollination process, i.e. pollinaria removal. Flies were collected from *C. boonjarasii* and *C. tenuicaulis* flowers. Those with pollinaria attached to the mouthparts were screened out. In addition to flies collected from flowers, we tested whether a laboratory strain of *Drosophila melanogaster*, which belongs to a completely different family from the identified pollinators or visitors and has never interacted with flowers (of *Ceropegia* or of other plants), could potentially be able to remove pollinaria.

Fully developed flower buds (recipient flowers) of *C. acicularis* (n = 7), *C. citrina* (n = 2) and *C. tenuicaulis* (n = 3) were bagged (mesh size 0.5 × 0.65 mm) to exclude any visitors. At anthesis, the target flies were gently dropped into each flower (one to eight fly individuals belonging to one to two fly species) using a moistened paintbrush, and the neck of the corolla tube was plugged with cotton to prevent insects from escaping and additional pollinators from entering. The number of species and individuals per species of fly introduced to a given flower was different depending on availability of insects obtained from flowers on each day of the experiment. Flies were kept inside the foreign flowers for 24 h, after which the manipulated flowers were collected in 70% ethanol. For each flower, the number of pollinaria removed from the anthers and the number of insects carrying pollinaria were counted in the laboratory.

Subsequently, the morphological structures of the gynostegia and the surrounding coronas of the four *Ceropegia* species were examined to inspect if there were any correlations with the result obtained from field experiments. Moreover, to have a broad perspective of the existence of mechanical isolation due to morphological fit between male and female floral parts, the microstructure of pollinaria, particularly the insertion crests, was also explored. These observations were made under light microscopes.

### Morphometric analyses of flowers and flies

To evaluate the differences between plant species in quantitative floral morphological traits that may function to restrict access by insects, we measured the following characters on 10 individuals (one flower per individual) of *Ceropegia acicularis* and *C. tenuicaulis*, on seven individuals (three of which had two flowers each) for *C. boonjarasii*, and six individuals (two of which with two and three flowers, respectively) for *C. citrina*: total flower length, corolla tube length, smallest diameter of corolla tube (i.e. the constriction of the tube above the basal inflation in *C. boonjarasii* and *C. citrina* and the small openings at the topmost part of the corolla tube in *C. acicularis* and *C. tenuicaulis*), basal inflation length and widest diameter of basal inflation. For the flowers collected from the same individual, mean values were used. In addition, the body length and width of floral visitors were measured.

Interspecific differences in the measured traits of flowers and of flies were tested using multivariate analysis of variance (MANOVA) with the five floral characters as dependent variables and plant species as the main factor; and with the length and width as dependent variables and dipteran morphospecies as main factor. A principal components analysis (PCA) was conducted to evaluate to what extent the measured traits of flowers differed between the studied species. Also, in order to test which floral traits were different among the studied species, univariate linear models (LM) were performed using each of the five measured floral traits as response variables and the plant species as categorical variables. Moreover, the correlation between the body length and width of insects and the values measured for the smallest diameter of corolla tube, the critical trait that could function as a morphological filter restricting Diptera access to flowers, was investigated by a regression analysis with Holm-adjusted p-values.

### Analyses of floral volatiles

Volatile organic compounds (VOCs) were collected from the flowers (one flower per plant individual) of *Ceropegia citrina* (n = 4), *C. tenuicaulis* (n = 6), *C. boonjarasii* (*n* = 7), and *C. acicularis* (n = 7) using dynamic headspace methods^63,64^ (for details see^34^). Flowers were enclosed in a polyethylene terephthalate (PET) bag for 30 min and sampled for 5 min. The cycle was repeated two times (thus 15 min sampling time and 90 min accumulation time) in *C. boonjarasii* and *C. citrina*. Samples were taken on the first day of anthesis. Control samples of ambient air were collected in parallel from empty PET bags. All samples were analyzed by gas chromatography coupled to mass spectrometry (GC–MS) at the ‘Platform for Chemical Analyses in Ecology’ (PACE, Montpellier) technical facilities of the LabEx CeMEB (‘Centre Méditerranéen pour l’Environnement et la Biodiversité,’ Montpellier, France) (see^34^).

Chemical data were processed using MZmine version 2.18^65^ adapted to GC data processing (customized software available on demand), using the same automated protocol for all samples in order to ensure consistency of peak integration. In order to clearly identify plant-emitted VOCs, all volatiles present in control samples of ambient air were omitted from downstream analyses. We discarded all the VOCs that were present in trace amount, meaning compounds that had per species a mean relative amount < 0.1%. None of these compounds was present in > 50% of individuals of a species. The retention times of a series of n-alkanes (standard solution of alkanes, 04070, Sigma Aldrich, Munich, Germany) were used for converting retention times to retention indices. Identity of VOCs was determined by comparing the mass spectra with those in the NIST database (NIST 2007 library; Wiley, 9th edition), and by comparing the calculated retention indices with those reported in the literature^66^. Moreover, when possible, the identity of VOCs was confirmed by comparison with mass spectra and retention times of authentic standards, using the same equipment and method. Non-Metric Multidimensional Scaling (NMDS) based on standardized relative proportions of VOCs was applied on flower samples, using the Bray–Curtis distance. Differences in floral volatiles among plant species were tested using PERMANOVA (10,000 permutations). Pairwise PERMANOVAs were run and p-values were adjusted for multiple comparisons using the FDR method. Indicator Species Analysis^67^ was applied to the VOCs (10,000 permutations) in order to identify those specific to each species.

### Attractive function of vibratile trichomes

In order to determine whether the vibratile trichomes on the corolla lobe tips play a role in attraction of pollinators to the flowers of *C. boonjarasii* (Fig. 3B), these trichomes were removed from flowers (n = 11; 5 plant individuals, 1–4 flowers per plant) and the number of flower-visiting Diptera was compared to those visiting unmanipulated flowers (n = 7; 5 plant individuals, 1–2 flowers per plant). To be sure that the two treatments would be exposed to floral visitors for the same period of time, the flower buds were enclosed in nylon bags (mesh size 0.5 × 0.65 mm) on the day prior to the experiment, and once opened the next morning, the trichomes were entirely removed (for manipulated flowers) and visitation was allowed. The trichomes were loosely attached to the surface of corolla lobes (most likely owing to their constricted base at the attachment point; see^29^) and thus could be easily pulled out by hand without damaging flowers. As all flowers did not open at the same time, the whole experiment was performed over four days. When possible (depending on the number of flowers that opened), for each day of the experiment, the two treatments were applied in parallel to a pair of flowers on the same plant or on different plants growing nearby in the same patch. At the end of the day, before the flowers wilted (for flower behavior upon withering of each studied species, see the “Results” section: Morphometric analyses of flowers and flies) and released the insects trapped inside^20,33^ flowers were collected in 70% ethanol and flies inside each flower were counted. The effect of experimental shaving was tested using a Generalized Linear Model (GLM) with number of trapped flies as the dependent variable and presence of vibratile trichomes and the individual plants as fixed factors.

All statistical analyses were performed with R 4.0.4^68^.

## Supplementary Information

Supplementary Information.

Supplementary Information 1.

Supplementary Information 2.

Supplementary Information 3.

Supplementary Video 1.

Supplementary Video 2.

Supplementary Video 3.

## Data Availability

All data generated or analysed during this study are included in this published article and its Supplementary Information files.
