# Supplementary material for: Pollinator and floral odor specificity among four synchronopatric species of Ceropegia (Apocynaceae) suggests ethological isolation that prevents reproductive interference
Source: Sci Rep. 2022 Aug 13;12:13788. doi: 10.1038/s41598-022-18031-z (PMC9376067; doi:10.1038/s41598-022-18031-z)
Supplement: Supplementary file 1 — Supplementary Information. [file 41598_2022_18031_MOESM1_ESM.docx]

**Supplementary Figures and Tables**

**Pollinator and floral odor specificity among four synchronopatric species of *Ceropegia* (Apocynaceae) suggests ethological isolation that prevents reproductive interference**

**Aroonrat Kidyoo^*^, Manit Kidyoo, Doyle McKey, Magali Proffit, Gwenaëlle Deconninck, Pichaya Wattana, Nantaporn Uamjan, Paweena Ekkaphan, Rumsaïs Blatrix**

^*^ aroonratm@hotmail.com

**Figure S1.** Maximum likelihood phylogenies of Milichiidae (A) and Chloropidae (B) flies pollinating four *Ceropegia* species from Pha Taem National Park *(C. acicularis*, *C. boonjarasii*, *C. citrina* and *C.* *tenuicaulis*) based on COI marker. Values on the trees are bootstrap values. GenBank accession numbers are in bold. Photos by Rumsaïs Blatrix.

**
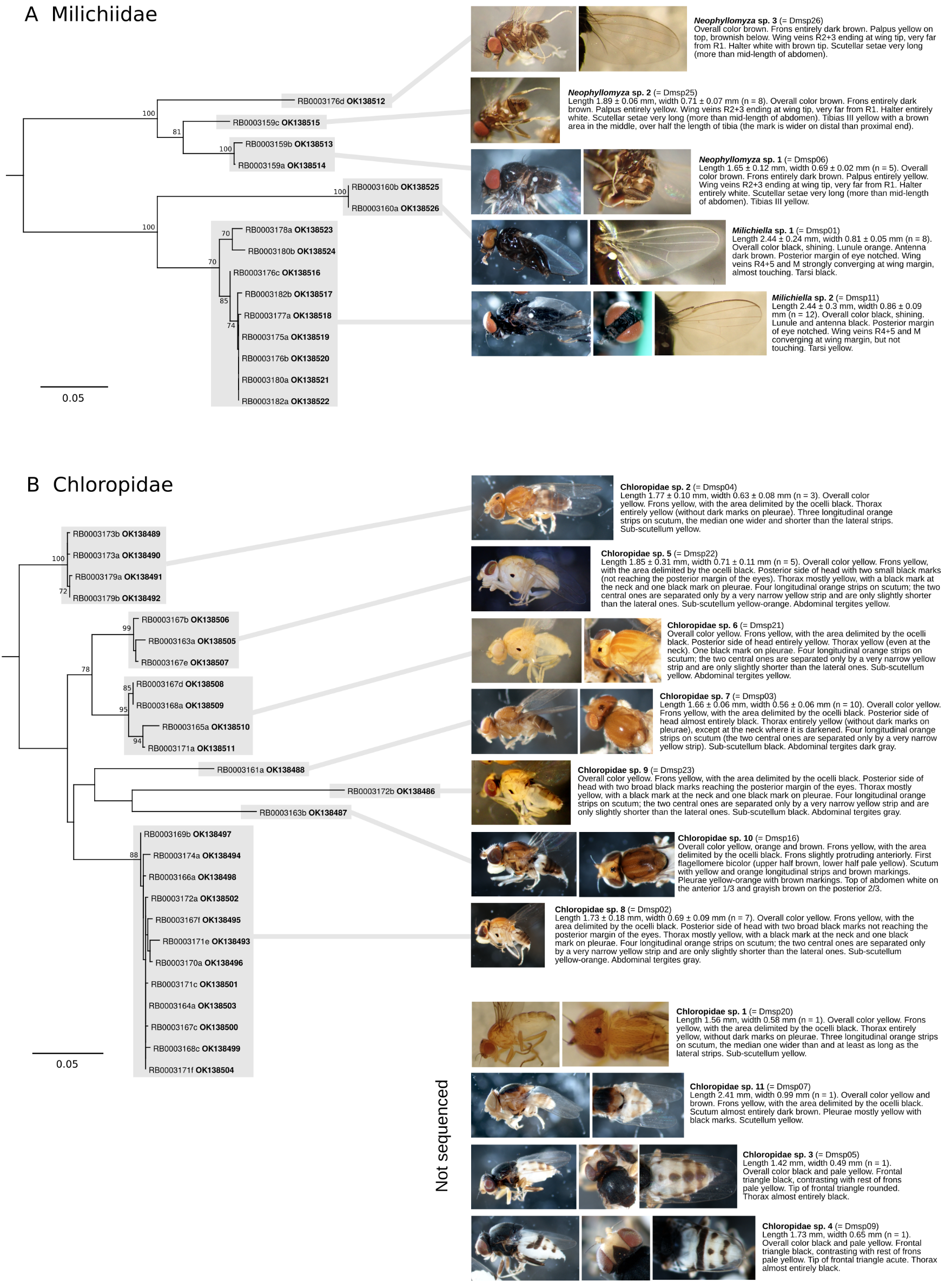
**

**
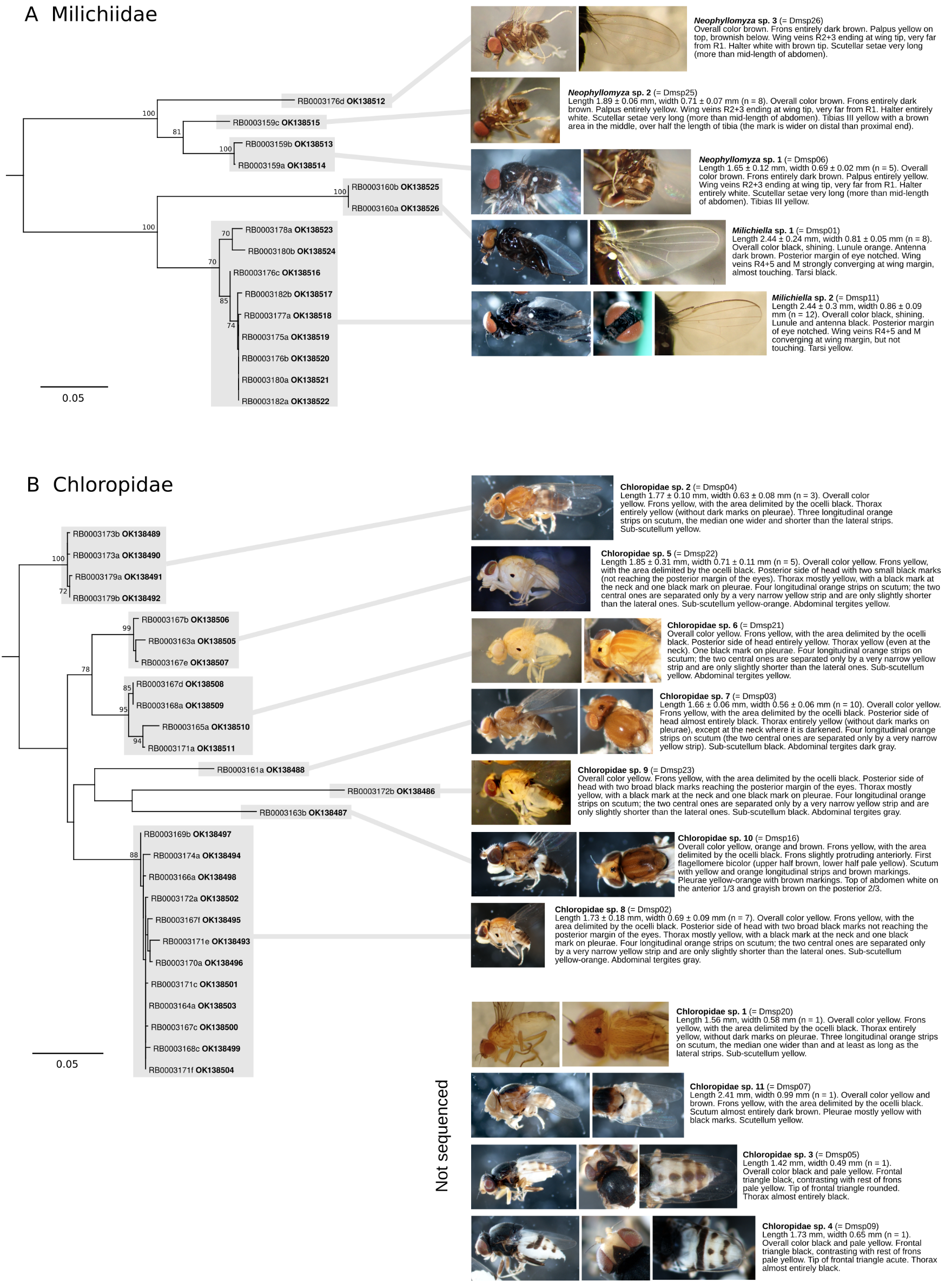
**

**Figure S2.** Visiting flies on *Ceropegia* flowers. (A) A Chloropidae fly on the top of the corolla tube of *C. acicularis* flower; (B) A Chloropidae fly near the window-like aperture of *C. boonjarasii* flower, heading towards the distal end of the flower where the flickering trichomes are found; (C) A flower of *C. citrina* with a *Neophyllomyza* fly landing on the inner side of the reflexed corolla lobe; (D) A flower of *C. tenuicaulis* with a *Milichiella* fly landing on the proximal portion of a corolla lobe.


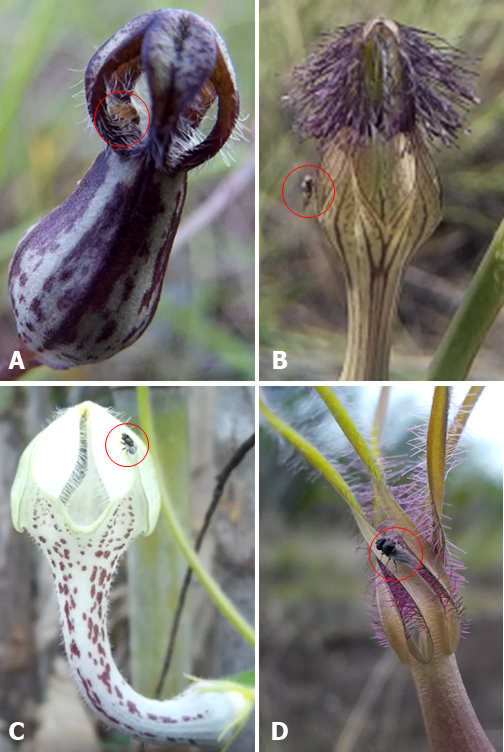


**Figure S3.** Scatter plot showing the body length and width of the individual fly pollinators of different morphospecies collected from flowers of the four studied *Ceropegia* species

**
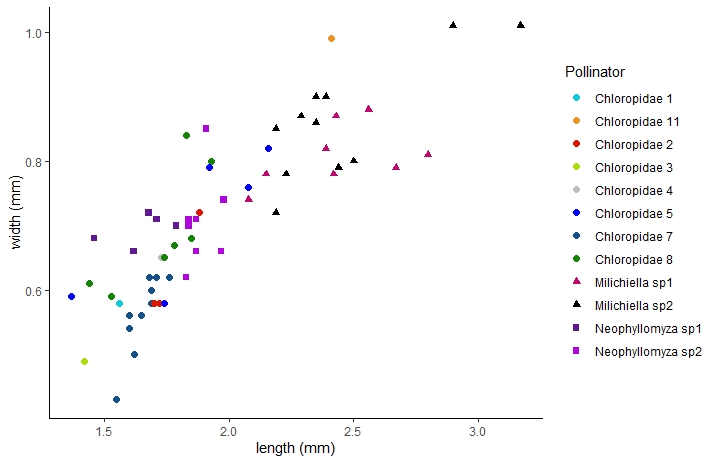
**

**Figure S4.** Presentation of study site.

(A) Map showing location of Pha Taem National Park. The park is located in the easternmost area of Thailand, with the Mekong River running alongside. It is distinctive for its sandstone mountains, having a surface area of 340 square kilometers, covered by dipterocarp and evergreen forests. The map was created using SimpleMappr (www.simplemappr.net).

(B) Distribution of individual plants of the four *Ceropegia* species in the main study area. Georeferencing of each individual allowed delineating patches for each species (*C. acicularis* in green, *C. boonjarasii* in orange, *C. citrina* in blue and *C. tenuicaulis* in pink). The map was created using Google Earth (www.earth.google.com)


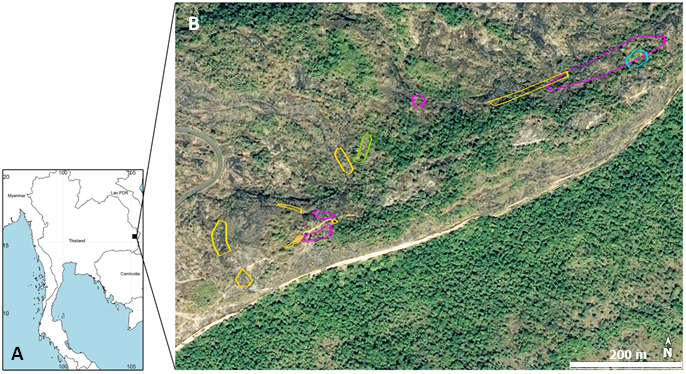


**Figure S5.** Study species.

(A) *Ceropegia acicularis* is an erect 10‒20 cm tall herb. It has simple terminal or subtermial flowers of yellowish- or pinkish-white color with reddish-brown strips or dots. The flower is constituted of a short straight urn-shaped corolla tube and five short lobes which are joined at tips.

(B) *Ceropegia boonjarasii* is a twining herb that produces one to two-flowered inflorescences in the leaf axils at 40‒200 cm height from the ground. Its funnel-shaped flower is yellowish- to greenish-white color with reddish-brown lines. The corolla tube is long straight upright and the corolla lobes are united at tips.

(C) *Ceropegia citrina* is a twining or prostrate herb bearing in the leaf axils the inflorescences that bear one to two flowers from ground level to 50 cm height. Its funnel-shaped flower is white to yellow with scattered reddish-brown or purple blotches. The corolla tube is strongly curved and the corolla lobes are fused at tips with the distal portion adorned with the clusters of long dark purple hairs.

(D) *Ceropegia tenuicaulis* is an erect 30–50 cm tall herb producing a terminal one-flowered inflorescence that bears greenish- or purplish-brown flowers. The flower has a long straight upright tubular to urn-shaped corolla tube. Its corolla lobes are linear and drawn into the long tails, loosely twisted at the base, but not joined at their tips.

Photos by Manit Kidyoo.

**
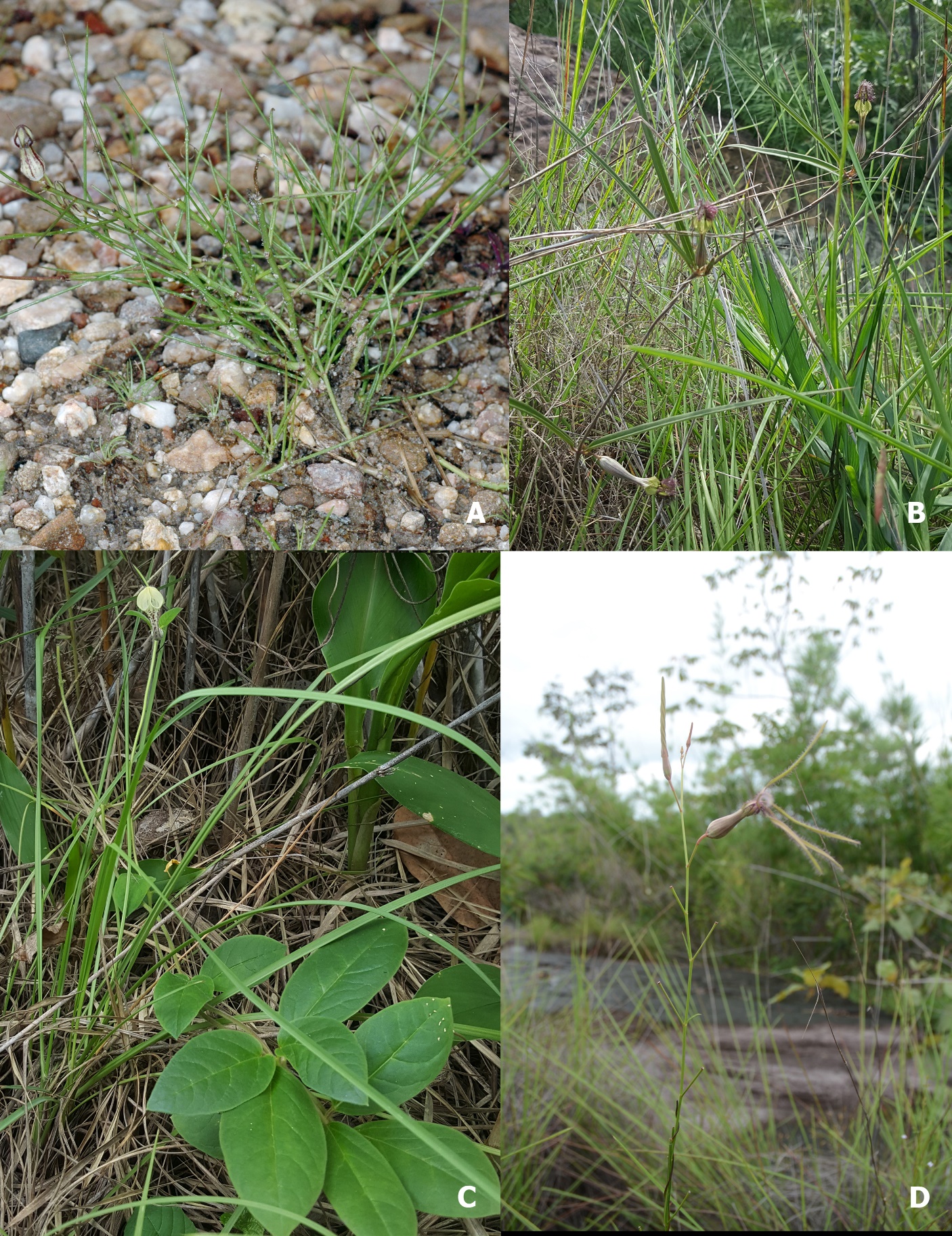
**

**Table S1.** Measurement of floral characters (mean ± SE; mm) compared between the four *Ceropegia* species

(A) Statistical difference for each floral trait measured.

| **Flower traits** | ***C. acicularis***  (n = 10) | ***C. boonjarasii***  (n = 7) | ***C. citrina***  (n = 6) | ***C. tenuicaulis***  (n = 10) | **Statistic difference between the 4 species** |
| --- | --- | --- | --- | --- | --- |
| total flower length | 12.3 ± 0.6 | 37.0 ± 1.1 | 42.9 ± 2.6 | 54.0 ± 2.2 | *F_3,29_* = 122.6, *P* < 0.001 |
| corolla tube length | 7.5 ± 0.4 | 24.5 ± 0.8 | 30.0 ± 1.5 | 17.1 ± 0.7 | *F_3,29_* = 142.2, *P* < 0.001 |
| smallest diameter of corolla tube | 2.6 ± 0.1 | 2.5 ± 0.1 | 4.2 ± 0.3 | 2.7 ± 0.1 | *F_3,29_* = 31.56, *P* < 0.001 |
| basal inflation length | 5.7 ± 0.3 | 12.8 ± 0.6 | 9.1 ± 0.3 | 9.7 ± 0.5 | *F_3,29_* = 42.85, *P* < 0.001 |
| widest diameter of basal inflation | 4.5 ± 0.2 | 6.2 ± 0.2 | 7.8 ± 0.4 | 5.9 ± 0.2 | *F_3,29_* = 30.04, *P* < 0.001 |

(B) Tukey multiple pairwise comparisons across *Ceropegia* species for each floral trait measured.

| **Flower traits** | **Pairs *Ceropegia* species compared** | **adjusted *P*** |
| --- | --- | --- |
| total flower length | *C. boonjarasii*-*C. acicularis* | < 0.001 |
|  | *C. citrina*-*C. acicularis* | < 0.001 |
|  | *C. tenuicaulis*-*C. acicularis* | < 0.001 |
|  | *C. citrina-C. boonjarasii* | 0.167 |
|  | *C. tenuicaulis*-*C. boonjarasii* | < 0.001 |
|  | *C. tenuicaulis*-*C. citrina* | < 0.001 |
| corolla tube length | *C. boonjarasii*-*C. acicularis* | < 0.001 |
|  | *C. citrina*-*C. acicularis* | < 0.001 |
|  | *C. tenuicaulis*-*C. acicularis* | < 0.001 |
|  | *C. citrina-C. boonjarasii* | < 0.001 |
|  | *C. tenuicaulis*-*C. boonjarasii* | < 0.001 |
|  | *C. tenuicaulis*-*C. citrina* | < 0.001 |
| smallest diameter of corolla tube | *C. boonjarasii*-*C. acicularis* | 0.984 |
|  | *C. citrina*-*C. acicularis* | < 0.001 |
|  | *C. tenuicaulis*-*C. acicularis* | 0.865 |
|  | *C. citrina-C. boonjarasii* | < 0.001 |
|  | *C. tenuicaulis*-*C. boonjarasii* | 0.715 |
|  | *C. tenuicaulis*-*C. citrina* | < 0.001 |
| basal inflation length | *C. boonjarasii*-*C. acicularis* | < 0.001 |
|  | *C. citrina*-*C. acicularis* | < 0.001 |
|  | *C. tenuicaulis*-*C. acicularis* | < 0.001 |
|  | *C. citrina-C. boonjarasii* | < 0.001 |
|  | *C. tenuicaulis*-*C. boonjarasii* | < 0.001 |
|  | *C. tenuicaulis*-*C. citrina* | 0.796 |
| widest diameter of basal inflation | *C. boonjarasii*-*C. acicularis* | < 0.001 |
|  | *C. citrina*-*C. acicularis* | < 0.001 |
|  | *C. tenuicaulis*-*C. acicularis* | < 0.001 |
|  | *C. citrina-C. boonjarasii* | < 0.001 |
|  | *C. tenuicaulis*-*C. boonjarasii* | 0.885 |
|  | *C. tenuicaulis*-*C. citrina* | < 0.001 |

**Table S2.** Body length and width of 12 (out of 14) identified dipteran morphospecies which were collected from flowers of the four studied *Ceropegia* species (see Table 1).

(A) Fly size (mean ± SE)

| **Fly species** | **Length (mm)** | **Width (mm)** |
| --- | --- | --- |
| *Milichiella* sp.1 (n = 8) | 2.44 ± 0.09 | 0.81 ± 0.02 |
| *Milichiella* sp.2 (n = 12) | 2.44 ± 0.09 | 0.86 ± 0.03 |
| *Neophyllomyza* sp.1 (n = 5) | 1.65 ± 0.06 | 0.69 ± 0.01 |
| *Neophyllomyza* sp.2 (n = 8) | 1.89 ± 0.02 | 0.71 ± 0.02 |
| Chloropidae sp. 1 (n = 1) | ca. 1.56 | ca. 0.58 |
| Chloropidae sp. 2 (n = 3) | 1.77 ± 0.06 | 0.63 ± 0.05 |
| Chloropidae sp. 3 (n = 1) | ca. 1.42 | ca. 0.49 |
| Chloropidae sp. 4 (n = 1) | ca. 1.73 | ca. 0.65 |
| Chloropidae sp. 5 (n = 5) | 1.85 ± 0.14 | 0.71 ± 0.05 |
| Chloropidae sp. 7 (n = 10) | 1.66 ± 0.02 | 0.56 ± 0.02 |
| Chloropidae sp. 8 (n = 7) | 1.73 ± 0.07 | 0.69 ± 0.04 |
| Chloropidae sp. 11 (n =1) | ca. 2.41 | ca. 0.99 |

(B) The correlation between the body length and width of insects and the values measured for smallest diameter of corolla tubes of the studied *Ceropegia* species was investigated by a regression analysis with Holm adjusted p-values. *R*^2^ and adjusted p-values are shown in the table. None of the models show a significant relationship.

| **Trait** | **smallest diameter of corolla tube** |
| --- | --- |
| fly length | 0.03 (*P* = 0.52) |
| fly width | 0.0002 (*P* = 0.91) |
| length x width | 0.02 (*P* = 0.62) |
