# Supplementary material for: Pollinator and floral odor specificity among four synchronopatric species of Ceropegia (Apocynaceae) suggests ethological isolation that prevents reproductive interference
Source: Sci Rep. 2022 Aug 13;12:13788. doi: 10.1038/s41598-022-18031-z (PMC9376067; doi:10.1038/s41598-022-18031-z)
Supplement: Supplementary file 2 — Supplementary Information 1. [file 41598_2022_18031_MOESM2_ESM.docx]

**Supplementary Information S1**

**Pollinator and floral odor specificity among four synchronopatric species of *Ceropegia* (Apocynaceae) suggests ethological isolation that prevents reproductive interference**

**Aroonrat Kidyoo^*^, Manit Kidyoo, Doyle McKey, Magali Proffit, Gwenaëlle Deconninck, Pichaya Wattana, Nantaporn Uamjan, Paweena Ekkaphan, Rumsaïs Blatrix**

^*^ aroonratm@hotmail.com

**Supplementary information S1.** Description of floral anthesis and pollinator behavior based on video recordings.

Flowers of all *Ceropegia* species frequently opened at daybreak, soon after the first appearance of sunlight, at about 06:00. The flowers were erect and the proximal portions of the corolla lobes split open, then the fissures widened until the five window-like apertures were fully opened, and for the species with tufts of long hairs on the top flower, i.e. *C. boonjarasii*, the hairs were soon wholly spread out. These processes generally took several dozen minutes. Anthesis of a single flower often lasted only one or rarely two days. After anthesis, the flowers of all *Ceropegia* species except *C. acicularis* bent down, mostly during nighttime or less frequently in the afternoon, releasing the insects trapped inside the flowers. It is noticeable that the post-anthesis flowers of *C. acicularis* never bent down, but only stopped emitting floral scents, and the trichomes around the window-like apertures became withered. When in full bloom, based on human perception, the flower of *C. tenuicaulis* emitted a very strong musty smell like that of dirty socks. The blooming flowers of *C. acicularis* released a less potent odor reminiscent of baby spit-up. The floral odor of *C. citrina* was very faint, hardly detectable by the human nose. Likewise, the flowers of *C. boonjarasii* were virtually odorless.

Video recordings showed Diptera, Hymenoptera and spiders (order Araneae) on *Ceropegia* flowers. Spiders often caught flies visiting flowers. On some plant individuals, ants patrolled flowers and the vicinity, and sometimes walked into and out of the flowers. The presence of ants usually disturbed visitation of flies, causing a low number of visits or even no visits to these flowers.

The Chloropidae flies that were the major pollinators of *C. boonjarasii* mostly landed on the corolla tube, then moved upward to the direction of the vibratile trichomes that were vigorously fluttering in the wind and reflecting the sunlight at dusk (Fig. S2B). When they passed through the inner side of the incurved corolla lobes (Fig. S2B), they very often slipped to the inside of the corolla tube through the window-like orifices.

In *Ceropegia citrina*, the *Neophyllomyza* flies usually landed on the inner side of the reflexed corolla lobes (Fig. S2C) or perched on the lower edge of the window-like orifices and often slipped and fell into the curved corolla tube shortly after landing. Most individuals of the *Milichiella* flies visiting the flowers of *C. tenuicaulis* landed on the proximal corolla lobes (Fig. S2D) or less frequently on the rim of the orifices. As the whole flower waved and the very long corolla lobes fluttered in the wind, the flies then fell right into the straight corolla tube.

The behavior of Chloropidae flies when visited the flower of *C. acicularis* can be clearly seen from Video 1. The behavior of *Milichiella* flies held inside the basal inflated portion of the flowers of *C. tenuicaulis* was revealed in Video 2. Moreover, Video 3 showed the behavior of a Chloropidae fly, with a pollinarium attached to its mouthpart, within the flowers of *C. acicularis.*
