# Supplementary material for: Pollinator and floral odor specificity among four synchronopatric species of Ceropegia (Apocynaceae) suggests ethological isolation that prevents reproductive interference
Source: Sci Rep. 2022 Aug 13;12:13788. doi: 10.1038/s41598-022-18031-z (PMC9376067; doi:10.1038/s41598-022-18031-z)

Simplified identification key for the morphospecies of Chloropidae and Milichiidae found in flowers of four *Ceropegia* species in Pha Taem National Park, Thailand

Aroonrat Kidyoo, Manit Kidyoo, Doyle McKey, Magali Proffit, Gwenaëlle Deconninck, Pichaya Wattana, Nantaporn Uamjan, Paweena Ekkaphan, Rumsaïs Blatrix. Pollinator and floral odor specificity among four synchronopatric species of Ceropegia (Apocynaceae) suggests ethological isolation that prevents reproductive interference. Scientific Reports.

1

Thorax in side view mostly black or dark brown 2

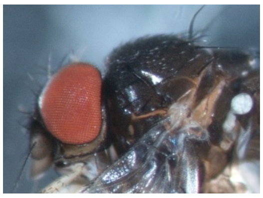
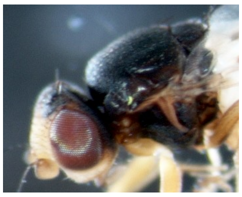

Thorax in side view mostly yellowish or light beige 8

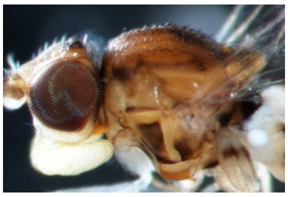
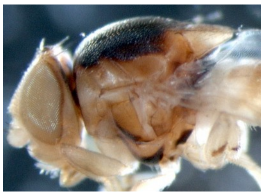

2

Posterior margin of eye with a notch 3

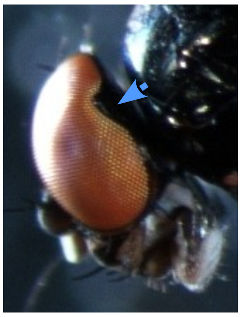

Posterior margin of eye straight, without a notch 4

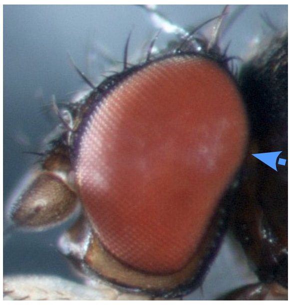

3

Tarsi black. Lunule orange. Wing veins R4+5 and M almost touching at wing margin .... ***Milichiella* sp. 1 (= Dmsp01)**

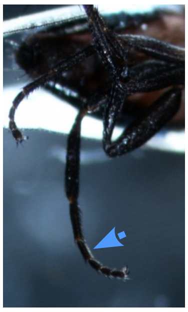
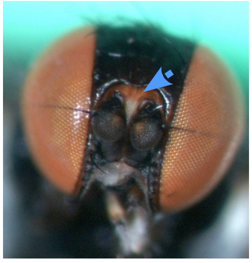
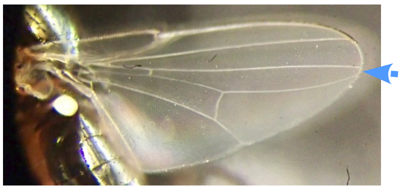

Tarsi yellow. Lunule black. Wing veins R4+5 and M convergent but clearly distant from each other at wing margin .... ***Milichiella* sp. 2 (= Dmsp11)**

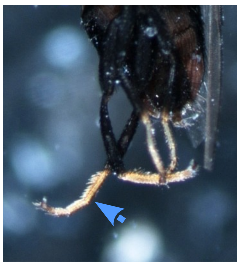
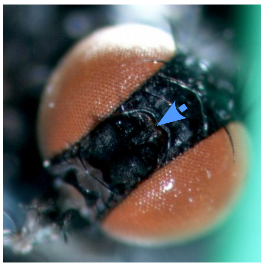
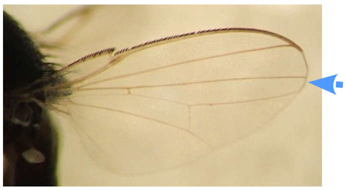

4

Frons entierly black, concolorous with ocellar triangle 5

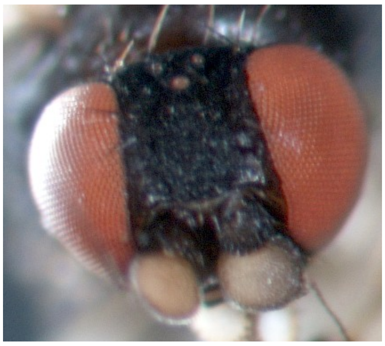

Frons yellow, contrasting with black ocellar triangle 7

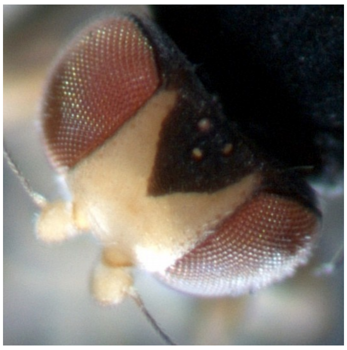

5

Tip of halter brown. Palpus yellow on upper side and light brown on lower side .... ***Neophyllomyza* sp. 3 (=Dmsp26)**

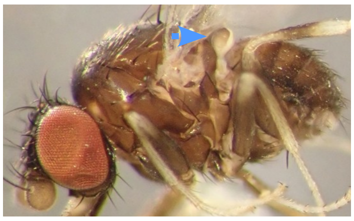

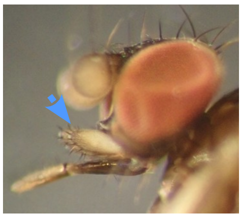

Halter entierly white. Palpus entierly yellow 6

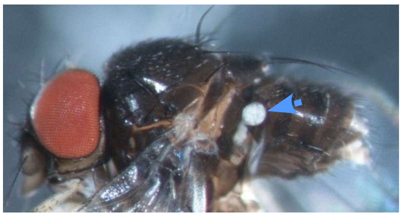

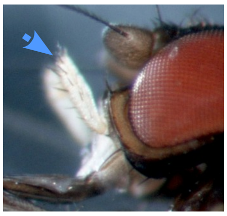

6

Tibia III yellow, sometimes very slightly darkened in the middle .... ***Neophyllomyza* sp. 1 (= Dmsp06)**

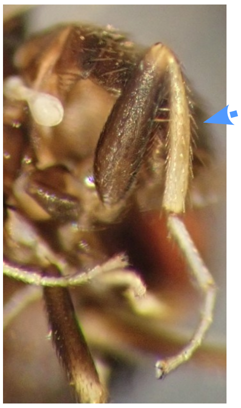

Tibias III yellow with a brown spot in the middle, over half the length of tibia on upper side, less extended on lower side .... ***Neophyllomyza* sp. 2 (=Dmsp25)**

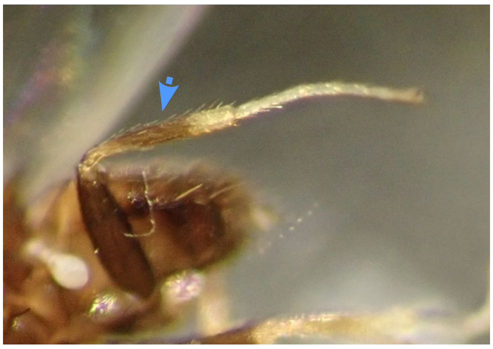

7

Anterior tip of ocellar triangle rounded. Abdomen pale yellow, with brown spots on median line and on lateral margins of tergites .... **Chloropidae sp. 3 (= Dmsp05)**

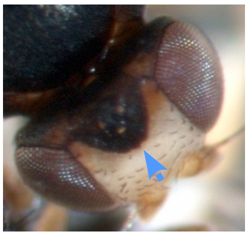

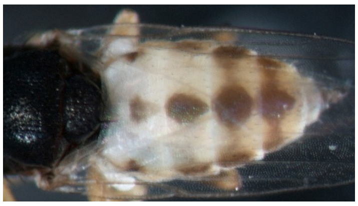

Anterior tip of ocellar triangle accute. Abdomen white, with black spots on lateral margins of tergie 1, tergite 2 entierly black, and tergites 3 and 4 with three black spots fused through a transverse black strip more or less thick ....**Chloropidae sp. 4 (= Dmsp09)**

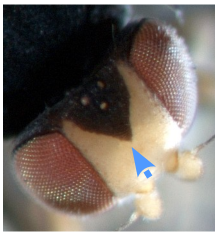

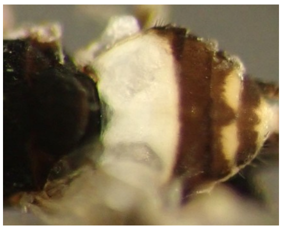

8

Thorax entirely yellow (or orange).. 9

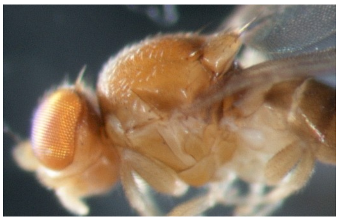
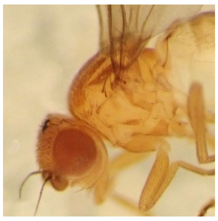

Thorax in side view with at least one brown or black spot 11

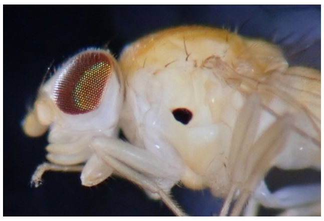
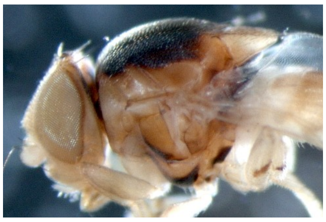

9

Subscutellum black. Posterior side of head entierly black, thorax black at the level of the junction with head .... **Chloropidae sp. 7 (= Dmsp03)**

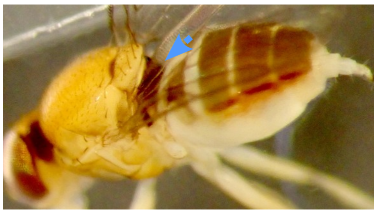
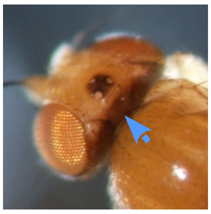

Subscutellum yellow. Posterior side of head and thorax yellow 10

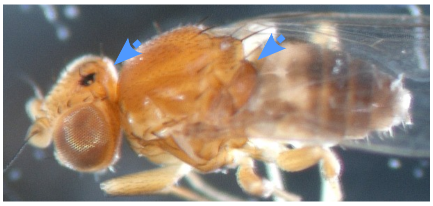

10

The median orange strip on suctum is shorter than the two lateral strips .... **Chloropidae sp. 2 (= Dmsp04)**

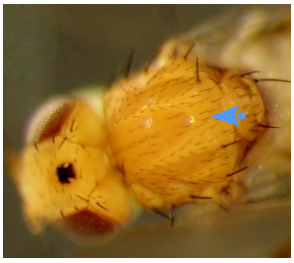

The median orange strip on suctum is at least as long as the two lateral strips .... **Chloropidae sp. 1 (= Dmsp20)**

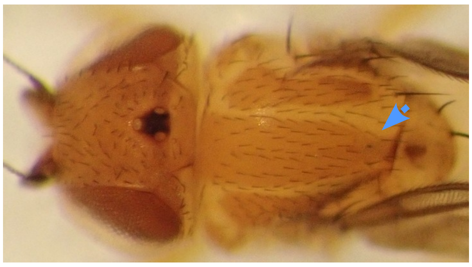

11

Scutum entierly yellow with 4 longitudinal orange strips. Thorax in side view only with a single small black spot in the middle 12

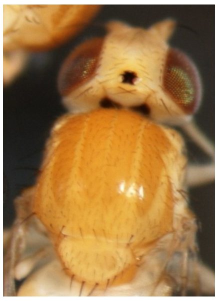
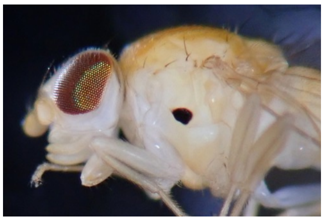

Scutum with brown or black parts. Thorax in side view with more than one small brown or black spot 15

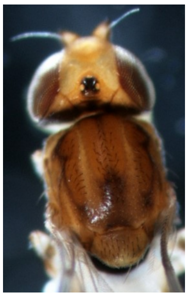
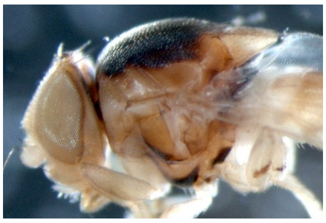

12

No black part at the junction of thorax on head .... **Chloropidae sp. 6 (= Dmsp21)**

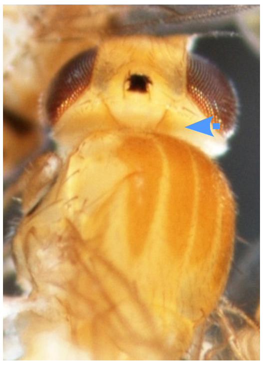

Black part at the junction of thorax on head 13

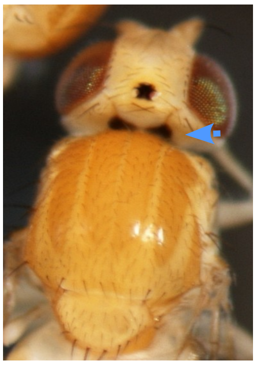

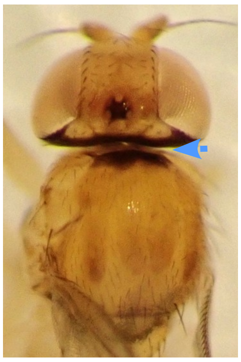

13

Suscutellum black. The black part on posterior side of head is wide and reaches the eyes .... **Chloropidae sp. 9 (= Dmsp23)**

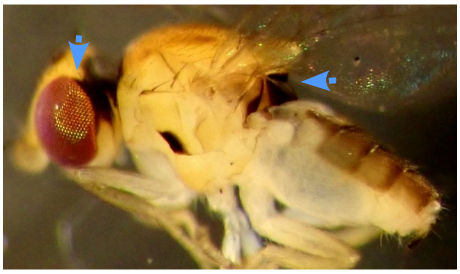

Suscutellum yellow. The black part on posterior side of head does not reach the eyes 14

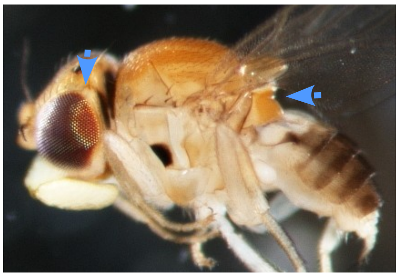

14

Two large black spots at the posterior side of heade. Abdomen grey .... **Chloropidae sp. 8 (= Dmsp02)**

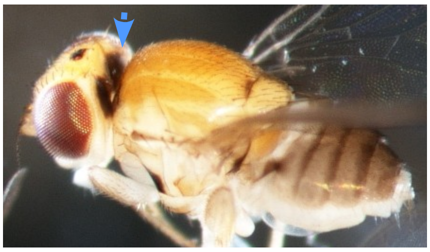

Two small black spots at the posterior side of head. Abdomen yellow .... **Chloropidae sp. 5 (= Dmsp22)**

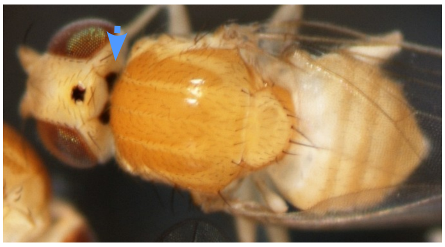

15

Scutum with brown and orange parts. First abdominal tergite white, the next three tergites entierly brown-dark grey.. **Chloropidae sp. 10 (= Dmsp16)**

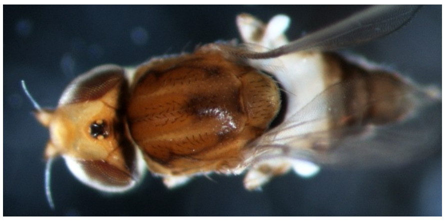

The anterior 2/3 of scutum black. The posterior 1/3 yellow. Abdomen yellow with darker spots .... **Chloropidae sp. 11 (= Dmsp07)**

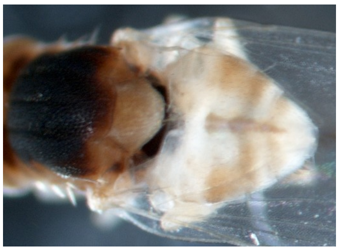

Supplement: Supplementary file 3 — Supplementary Information 2. [file 41598_2022_18031_MOESM3_ESM.pdf]
