# Supplementary material for: Pollinator and floral odor specificity among four synchronopatric species of Ceropegia (Apocynaceae) suggests ethological isolation that prevents reproductive interference
Source: Sci Rep. 2022 Aug 13;12:13788. doi: 10.1038/s41598-022-18031-z (PMC9376067; doi:10.1038/s41598-022-18031-z)
Supplement: Supplementary file 4 — Supplementary Information 3. [file 41598_2022_18031_MOESM4_ESM.docx]

**Supplementary Information S3**

**Pollinator and floral odor specificity among four synchronopatric species of *Ceropegia* (Apocynaceae) suggests ethological isolation that prevents reproductive interference**

**Aroonrat Kidyoo^*^, Manit Kidyoo, Doyle McKey, Magali Proffit, Gwenaëlle Deconninck, Pichaya Wattana, Nantaporn Uamjan, Paweena Ekkaphan, Rumsaïs Blatrix**

^*^ aroonratm@hotmail.com

**Supplementary information S3.** List of vouchers specimens of each studied *Ceropegia* species.

*C. acicularis* Kidyoo: THAILAND. Ubon Ratchathani: Pha Morn, Pha Taem National Park, 240 m, 13 July 2016, *M. Kidyoo 1645* (BCU)

*C. boonjarasii* Kidyoo: THAILAND. Ubon Ratchathani: Pha Morn, Pha Team National Park, 240 m, 5 July 2017, *M. Kidyoo 1648* (BCU)

*C. citrina* Kidyoo & A. Kidyoo: THAILAND. Ubon Ratchathani: Pha Morn, Pha Taem National Park, 240 m, 2 August 2017, *M. Kidyoo 1651* (BCU)

*C. tenuicaulis* Kidyoo: THAILAND. Ubon Ratchathani: Pha Morn, Pha Taem National Park, 240 m, 5 July 2017, *M. Kidyoo 1647* (BCU).
